# A Developmental Neuroimmune Cascade Model of Autism Spectrum Disorder

**DOI:** 10.3390/ijms27125185

**Published:** 2026-06-08

**Authors:** Gerry Leisman, Robert Melillo, Rahela Alfasi

**Affiliations:** 1 Movement and Cognition Laboratory, Department of Physical Therapy, University of Haifa, Haifa 3498838, Israel; drm@drrobertmelillo.com (R.M.); rahela.alfasi@gmail.com (R.A.); 2 Resonance Therapeutics Laboratory, University of the Medical Sciences of Havana, Havana 10400, Cuba; 3 Center for Developing Minds, Rockville Centre, NY 11570, USA

**Keywords:** autism, neuroimmunity, immune dysregulation, brain, insula, functional connection, maturation, neurotransmitters

## Abstract

Autism spectrum disorder (ASD) is a heterogeneous neurodevelopmental condition characterized by complex interactions among genetic, environmental, and biological factors. Increasing evidence suggests that immune system processes intersect with neurodevelopment in ways that may influence brain maturation, synaptic organization, and large-scale network function. However, existing literature is often fragmented across molecular, cellular, and systems levels, limiting the development of a coherent interpretive framework. In this review, we propose a developmental neuroimmune cascade model of ASD, in which early-life immune perturbations, arising from prenatal or perinatal factors, may interact with genetic susceptibility to influence cytokine signaling, microglial function, blood-brain barrier dynamics, and gut-immune communication. These processes may, in turn, affect synaptic pruning, excitatory-inhibitory balance, and the maturation of neural circuits, contributing to alterations in large-scale brain networks implicated in sensory processing, interoception, and social cognition. We synthesize evidence from observational human studies, postmortem analyses, and experimental animal models to examine how immune-related mechanisms may contribute to neurodevelopmental trajectories associated with ASD, while explicitly distinguishing associative findings from mechanistic inference. Particular attention is given to the role of distributed network vulnerability, including, but not limited to, insula-centered systems that integrate internal bodily states with affective and cognitive processing. Finally, we consider implications for biomarker development and stratified intervention approaches, emphasizing the importance of developmental timing, biological heterogeneity, and cautious interpretation of translational potential. Rather than positioning immune dysfunction as a singular cause of ASD, this model conceptualizes neuroimmune processes as modulators of developmental trajectories, offering a structured basis for future research linking immune signaling to circuit-level and behavioral outcomes.

## 1. Introduction

Autism spectrum disorder (ASD) is a complex neurodevelopmental condition characterized by marked heterogeneity in clinical presentation, cognitive profiles, sensory processing, and developmental trajectories [1,2]. While ASD has traditionally been conceptualized primarily as a disorder of brain development, increasing attention has been directed toward the role of immune system processes as contributors to neurodevelopmental variation associated with the condition [3,4]. Converging evidence from genetic, molecular, and physiological studies indicates that immune signaling intersects with neurodevelopment across early life, suggesting that immune-related mechanisms may influence the maturation of neural systems rather than operate as isolated pathological drivers. In this sense, timing may function as an organizing principle influencing neural dynamics that constrains the coordination, synchronization, and integration of neural activity across developmental timescales. This review advances a specific, testable developmental hypothesis. We propose that early motor timing and coordination may function as important organizing constraints on neural development, shaping the temporal structure within which higher-order cognitive and social functions emerge. Within this framework, disruptions in early timing mechanisms may precede and predict later impairments in social coordination, interoceptive processing, and cognitive integration. This formulation allows the proposed neuroimmune cascade model to be evaluated empirically by linking early motor variability, immune signaling profiles, and longitudinal network-level outcomes.

A growing body of research has focused on neuroinflammatory processes, including altered activation states of microglia and other immune-related cells within the central nervous system, in individuals with ASD [3,5]. These processes have been associated with differences in synaptic development, neural connectivity, and circuit maturation. At the same time, peripheral immune interfaces, particularly the intestinal epithelial barrier, have been implicated in shaping systemic immune signaling and its potential effects on brain development [6,7]. Altered gut barrier function may influence intestinal permeability and immune-brain communication pathways through interactions involving immune mediators, microbial metabolites, and circulating antigens.

Advances in genomic, transcriptomic, proteomic, and metabolomic approaches have further highlighted the complexity of immune involvement in ASD. Dysregulation of immune-related genes and signaling pathways has been identified in subsets of individuals, pointing to interactions between genetic susceptibility and immune function [8,9]. In parallel, environmental and maternal factors that alter immune activity during critical developmental periods, including maternal immune activation, infection, and metabolic conditions, have been associated with altered neurodevelopmental outcomes in offspring [10,11,12]. Together, these findings underscore the importance of developmental timing, as immune perturbations occurring during sensitive windows may exert long-lasting effects on neural circuit organization.

Despite substantial progress, the literature remains fragmented across levels of analysis. Immune-related findings are often examined independently of broader developmental processes, and associations between neuroimmune dysregulation and neural outcomes are frequently interpreted without a unifying developmental interpretive framework. Moreover, variability in ASD-associated developmental trajectories and biological profiles complicates efforts to identify consistent immune signatures, as findings differ across individuals, developmental stages, environmental exposures, and methodological approaches [13,14].

Current therapeutic approaches further reflect this gap, as most interventions target behavioral manifestations rather than underlying biological mechanisms, and immune-targeted strategies remain limited by incomplete mechanistic understanding and variable clinical efficacy [15].

In response to these challenges, the present review proposes a developmental neuroimmune cascade model of ASD. In this framework, early-life immune perturbations, arising from genetic susceptibility, environmental exposure, or maternal factors, may interact with cytokine signaling pathways, microglial function, and peripheral immune interfaces to influence processes such as synaptic pruning, excitatory-inhibitory balance, and large-scale network maturation. These interactions are conceptualized as temporally structured and developmentally dependent, rather than static or uniform across individuals.

Importantly, this model distinguishes between levels of evidence, integrating findings from human studies, postmortem analyses, and animal models while avoiding direct inference of causality where mechanistic support is limited. Rather than positioning immune dysfunction as a singular cause of ASD, the framework conceptualizes immune processes as modulators of developmental trajectories that may contribute to network-level vulnerability in specific biological and developmental contexts.

Within this broader perspective, particular attention is given to large-scale brain networks involved in interoception, sensory integration, and cognitive-affective processing. The insula and related salience-network structures are considered candidate integrative hubs that may be particularly sensitive to immune-related signals affecting the coordination of internal bodily states with higher-order processing. However, these regions are interpreted as part of a distributed network architecture, rather than as uniquely privileged loci of dysfunction, reflecting the likelihood that neuroimmune influences operate across multiple interconnected systems.

By organizing existing evidence into a coherent developmental cascade linking immune signaling to circuit-level and network-level outcomes, this review aims to provide a structured framework for understanding the neuroimmune contributions to ASD. This approach is intended to support future research focused on identifying mechanistic pathways, clarifying sources of heterogeneity, and informing the development of biologically grounded, developmentally sensitive strategies for investigation and intervention.

It is important to clarify that the role of motor timing proposed here is not merely correlational. While bidirectional interactions between motor and cognitive systems are well established, developmental evidence suggests an asymmetry in which early motor coordination constrains the temporal structure within which higher-order processes emerge. Thus, motor timing may function as an important organizing scaffold that shapes, rather than simply reflects, the development of cognitive and social functions. Understanding timing as a causal organizing principle has significant implications for early identification, intervention, and the design of developmentally informed learning environments. This raises a fundamental problem of developmental self-organization: how early biological signals constrain the emergence of coherent neural and behavioral structure across scales. Importantly, complex neurodevelopmental phenotypes may emerge either from interacting multifactorial processes or from single developmental perturbations capable of producing broad downstream developmental effects.

## 2. Literature Search and Selection Strategy

This review was conducted using a structured, though non-systematic, literature search strategy designed to capture recent and conceptually relevant work across neurodevelopment and neuroimmunology in autism spectrum disorder (ASD). Electronic databases, including PubMed, Scopus, and Web of Science were searched for articles published between 2010 and 2026, with particular emphasis on studies from the past five years to ensure contemporary relevance.

Search terms included combinations of the following keywords: autism, ASD, motor development, motor timing, primitive reflexes, neurodevelopmental asymmetry, insula, neuroimmunology, microglia, cytokines, connectivity, and developmental cascade. Boolean operators (AND, OR) were used to refine searches and identify intersecting domains (e.g., “ASD AND motor timing AND immune function”).

Inclusion criteria prioritized (i) peer-reviewed empirical studies, (ii) meta-analyses and systematic reviews, and (iii) high-quality theoretical or integrative papers directly relevant to developmental relationships among motor function, neural connectivity, and immune processes. Studies focusing on adult-only populations, unrelated neurological conditions, or lacking clear relevance to ASD developmental pathways were excluded unless they provided foundational mechanistic insights. Given the conceptual and integrative nature of the present review, priority was given to studies that informed cross-domain interactions (e.g., interactions between motor and immune-related processes, neurodevelopmental timing, and network-level developmental organization), rather than exhaustive coverage of any single domain. Where appropriate, seminal earlier works were included to provide theoretical grounding, while recent literature (2021–2026) was emphasized to reflect current empirical developments.

## 3. Genetic and Developmental Constraints on Neuroimmune Risk in ASD

### 3.1. Genetic Contributions to Neurodevelopmental and Neuroimmune Vulnerability

Autism spectrum disorder (ASD) is a highly heritable neurodevelopmental condition, with genetic factors accounting for a substantial proportion of individual risk [16,17]. Large-scale sequencing studies have identified numerous genes associated with ASD, many of which are involved in synaptic development, transcriptional regulation, chromatin remodeling, and neuronal differentiation [17,18]. These findings indicate that ASD does not arise from a single genetic pathway but rather reflects the cumulative effects of multiple variants influencing core developmental processes.

ASD-associated genetic variants typically exert modest effects on cellular function, particularly when compared with mutations observed in oncological conditions. Rather than producing overt cellular toxicity, these variants tend to alter developmental signaling pathways, neuronal differentiation, and synaptic organization [16]. Such effects may influence the timing and coordination of brain maturation, thereby shaping the conditions under which neural circuits form and stabilize. At the neural level, this process may reflect the progressive synchronization of oscillatory activity across thalamocortical and corticocortical networks, potentially supporting temporally coordinated communication between distributed regions and the emergence of relatively stable functional architectures.

In addition to genes directly involved in neuronal development, immune-related genes have been identified among ASD-associated risk loci. Variants influencing immune signaling, cytokine regulation, and microglial function suggest that immune pathways may interact with neurodevelopmental processes from early stages of brain maturation [19]. From this perspective, genetic architecture in ASD can be understood not only as specifying neural development, but also as modulating susceptibility to immune-related influences on that development. A summary of major gene categories and their functional roles is provided in Table 1.

### 3.2. Early Sensory Processing as a Developmental Interface

Neurodevelopmental alterations associated with ASD are frequently expressed through atypical sensory processing, now recognized as a core feature of the condition [20,21]. Individuals with ASD commonly exhibit sensory hyperresponsivity, hyporesponsivity, or sensory-seeking behaviors across modalities, reflecting differences in how sensory input is registered, integrated, and regulated. Developmental evidence suggests an asymmetry in which early motor coordination may constrain the temporal structure within which perceptual and cognitive systems emerge. Disruptions in these early timing mechanisms may therefore contribute to cascading effects on later-developing functions rather than simply reflecting downstream abnormalities.

Experimental studies in animal models have demonstrated that dysfunction in peripheral mechanosensory neurons can produce tactile abnormalities and behavioral phenotypes relevant to ASD [22]. These findings suggest that early disruptions in sensory input may influence downstream neural development by altering patterns of activity-dependent signaling. Animal models further suggest that altered sensory processing may influence social behavior, reinforcing the role of early sensory experience in guiding neurodevelopmental trajectories [23].

Human neuroimaging studies have similarly identified atypical neural responses within sensory and salience-related networks in ASD, including altered activation and connectivity in regions responsible for integrating sensory, affective, and attentional processes [24,25]. These findings suggest that early differences in sensory processing may interact with developing neural circuits, potentially amplifying or constraining the effects of other biological influences, including immune signaling.

### 3.3. Network-Level Outcomes of Genetic and Developmental Variation

The convergence of genetic susceptibility and early developmental processes in ASD is reflected in differences in the organization of large-scale brain networks. Variants affecting synaptic function, neuronal connectivity, and immune signaling may collectively influence the maturation of neural circuits, contributing to variability in functional connectivity and network integration across development [17,18].

Importantly, these network-level alterations are heterogeneous and developmentally dynamic. Differences in sensory, cognitive, and social functioning may arise from distinct combinations of genetic variation, developmental timing, and environmental exposure. This variability supports a model in which ASD emerges from interacting influences on brain maturation rather than from a single pathogenic mechanism. Within the framework advanced in this review, these genetic and developmental factors are conceptualized as shaping the developmental context by which neuroimmune processes operate. That is, genetic architecture and early sensory-driven activity shape the susceptibility of developing neural circuits to immune-related influences, including cytokine signaling, microglial activity, and peripheral immune interactions. In this way, genetic and neurodevelopmental variation likely interact with immune processes and may help define the context in which neuroimmune mechanisms may contribute to circuit-level and network-level outcomes. Recent transcriptomic analyses further indicate that immune-related gene expression is altered in the brains of individuals with neurodevelopmental disorders, supporting associations between genetic susceptibility and altered neuroimmune signaling pathways [26].

Evidence from genetic, sensory, and systems-level studies indicates that ASD reflects interactions among developmental pathways governing brain structure, connectivity, and function. These interactions provide a foundation for understanding how immune-related processes, introduced in the following sections, may influence neurodevelopmental trajectories in a temporally structured and biologically constrained manner.

## 4. The Insula and Salience Networks as Candidate Sites of Developmental Neuroimmune Vulnerability

### 4.1. Rationale for Considering the Insula Within a Neuroimmune Framework

**Figure 1:**
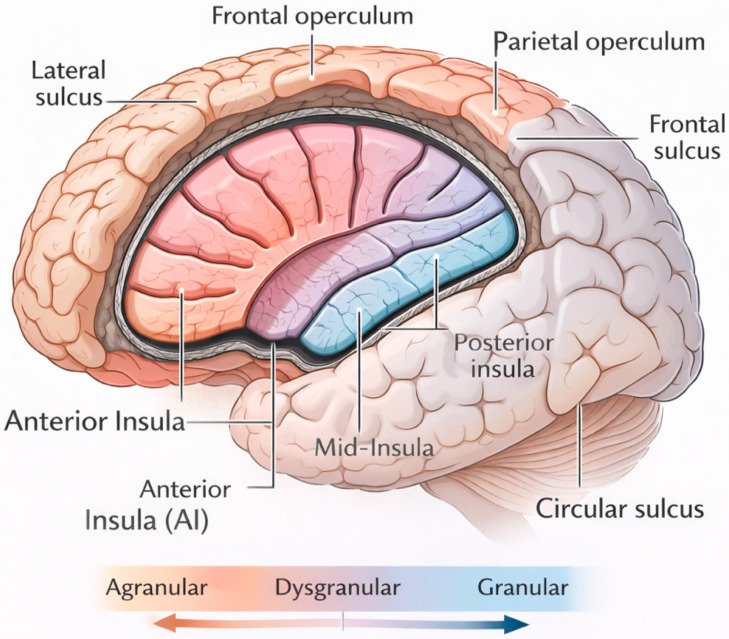
Anatomy of the human insular cortex. The human insular cortex is located bilaterally within the lateral sulcus, which separates the temporal lobe from the frontal and parietal lobes. It is covered by the frontal, parietal, and temporal opercula. The central insular sulcus divides the insula into anterior and posterior regions, while the circular sulcus delineates its perimeter. The anterior insula (AI) contains three short insular gyri, whereas the posterior insula (PI) contains two long insular gyri. Cytoarchitecturally, the insula comprises an anterior agranular region, a posterior granular region, and a transitional dysgranular midsection.

The insular cortex occupies a central position within distributed neural systems that integrate sensory, interoceptive, affective, autonomic, and cognitive information, as reflected in Figure 1. Anatomical studies have demonstrated extensive reciprocal connections between the insula and the anterior cingulate cortex, supporting coordinated processing of emotional, cognitive, and autonomic information [27,28]. The insula also maintains connections with primary and secondary somatosensory cortices, temporal regions, limbic structures, and prefrontal systems, positioning it as a convergence zone for bodily, emotional, and contextual signals [27].

Within the developmental neuroimmune framework proposed here, the insula is not treated as the sole or primary locus of ASD pathology. Rather, it is considered a candidate integrative hub within a broader distributed network vulnerability model. This distinction is important because immune-related influences on neurodevelopment are unlikely to affect one cortical region in isolation. Instead, cytokine signaling, microglial activity, blood-brain barrier dynamics, and peripheral immune-brain communication may influence the maturation of distributed circuits, including salience, interoceptive, sensory, affective, and executive systems.

### 4.2. Functional Connectivity, Interoception, and Salience Processing

Functional connectivity analyses indicate that the insula exhibits heterogeneous connectivity patterns across cerebral systems. Connectivity gradients extend along anterior-posterior and ventral-dorsal axes, linking anterior insular regions to higher-order transmodal networks, including prefrontal, anterior cingulate, and parietal systems [27,29]. These connections support attentional regulation, decision-making, and integration of internal bodily states with external environmental information.

Posterior insular regions are more closely associated with primary interoceptive and somatosensory representations, whereas anterior insular regions participate in higher-order affective, motivational, and cognitive integration. This posterior-to-anterior organization provides a developmental and functional basis for considering the insula as a site where bodily signals may be transformed into subjective feeling states, regulatory responses, and socially relevant affective information [28]. Within this framework, variability in motor timing may influence the precision of sensorimotor prediction and interpersonal synchrony. Instability in timing-related processes could affect the temporal coordination involved in gaze alignment, turn-taking, and affective mirroring, potentially influencing the development of social cognition and interpersonal interaction.

### 4.3. Emotion, Sensory Processing, and Developmental Timing

**Figure 2:**
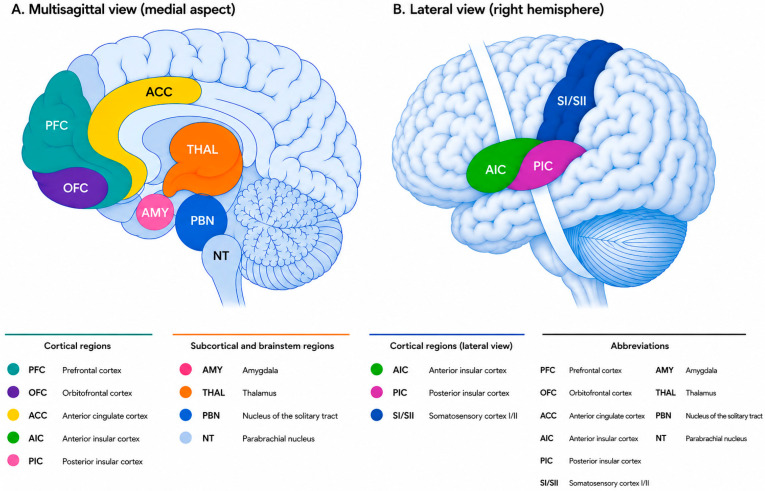
Central processing of interoceptive signals and their integration with emotional, cognitive, and motivational systems. Interoceptive information reflecting continuously changing bodily states ascends through spinal and brainstem pathways and reaches the posterior insular cortex (PIC) via specific thalamic relays. From the PIC, this information is projected rostrally to the anterior insular cortex (AIC), where it is integrated with emotional, cognitive, and motivational inputs from distributed cortical and subcortical regions. Through this integration, the anterior insula is proposed to support subjective feeling states and their incorporation into higher cognitive and motivational processes. The left panel depicts a midsagittal view of the human brain, highlighting regions involved in interoceptive processing. The right panel shows a lateral sagittal view emphasizing the insular cortex, including the AIC and PIC, as well as the primary and secondary somatosensory cortices (SI/SII). Subcortical processing involves the nucleus of the solitary tract (NTS), parabrachial nucleus (PB), and ventromedial thalamic nucleus, which project to the hypothalamus and insular cortex. Primary interoceptive signals from the thalamus project to the PIC, where integration with exteroceptive and proprioceptive information from SI/SII may occur. The AIC is strongly connected with paralimbic and prefrontal regions, including the orbitofrontal cortex (OFC), anterior cingulate cortex (ACC), and prefrontal cortex (PFC), supporting interactions between bodily states and affective-cognitive processes as well as descending regulatory signaling.

The insula’s involvement in emotional processing (as represented in Figure 2) has been demonstrated in neuroimaging studies examining responses to affective stimuli, including disgust. Both the experience and imagination of disgust activate overlapping regions within the anterior insula, suggesting a shared neural substrate for embodied emotional experience [30]. This convergence of sensory and affective processing supports the view that insular networks contribute to the transformation of bodily and sensory signals into emotionally meaningful states.

Developmental observations further emphasize the importance of interoceptive and affective systems in early social-emotional maturation. Around 18 months of age, facial expressions of disgust displayed by caregivers can elicit emotional responses such as guilt, shame, and embarrassment in the child, representing a developmental stage relevant to emerging social-emotional processing [31,32]. These processes have been associated with right-hemisphere maturation and broader social-emotional network development.

The insula also contributes to self-awareness, body ownership, and agency. Together with the anterior cingulate cortex, the right insula has been associated with self-referential processing and embodied self-awareness. Around the age of two years, children typically develop the ability to recognize themselves in mirrors or images, consistent with maturation of neural systems involved in self-awareness and agency [27]. These developmental milestones may relate to interoceptive processing and anterior insular network maturation.

**Figure 3:**
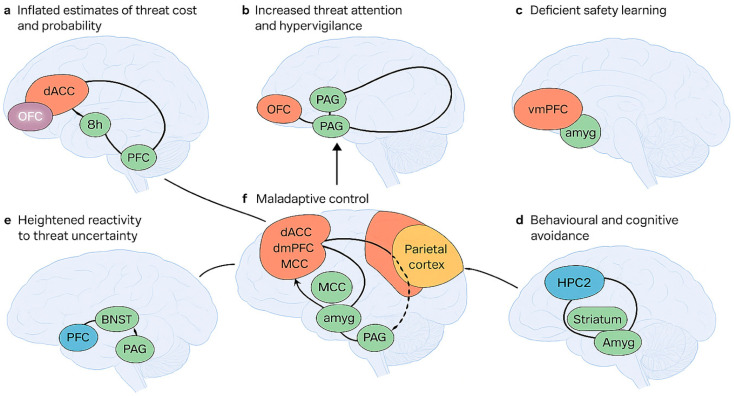
Neural circuitry associated with threat processing, uncertainty, and avoidance behavior. Altered threat probability and cost estimation are associated with activity within the dorsomedial prefrontal cortex (dmPFC), rostral cingulate cortex (rCing), orbitofrontal cortex (OFC), ventral striatum (VS), and anterior insula (AI). Increased amygdala (Amyg) activity may enhance basal forebrain (BF) modulation of sensory inputs, potentially heightening attention to threat-related stimuli. Interactions among the amygdala, OFC, and VS may further influence threat expectancy and attentional bias. Altered safety learning has been associated with differences in regulatory interactions between the ventromedial prefrontal cortex (vmPFC) and amygdala. Behavioral and cognitive avoidance are proposed to involve interactions between the amygdala and decision-making/action-selection networks involving the OFC, dorsolateral prefrontal cortex (dlPFC), striatum, anterior mid-cingulate cortex (aMCC), and AI [35].

Interoception, defined as the perception of internal bodily states, is relevant to empathy, affect regulation, theory of mind, and threat processing as reflected in Figure 3. Empirical evidence indicates that interoception, self-awareness, body ownership, agency, and empathy have been reported to be altered in individuals with ASD, including some nonspeaking autistic individuals [33,34]. These findings are consistent with the possibility that altered developmental timing and connectivity of insula-centered networks may contribute to specific ASD-related phenotypes, especially those involving sensory reactivity, affective regulation, uncertainty processing, and social-emotional integration.

### 4.4. Insula-Cingulate Maturation and ASD-Related Network Vulnerability

**Figure 4:**
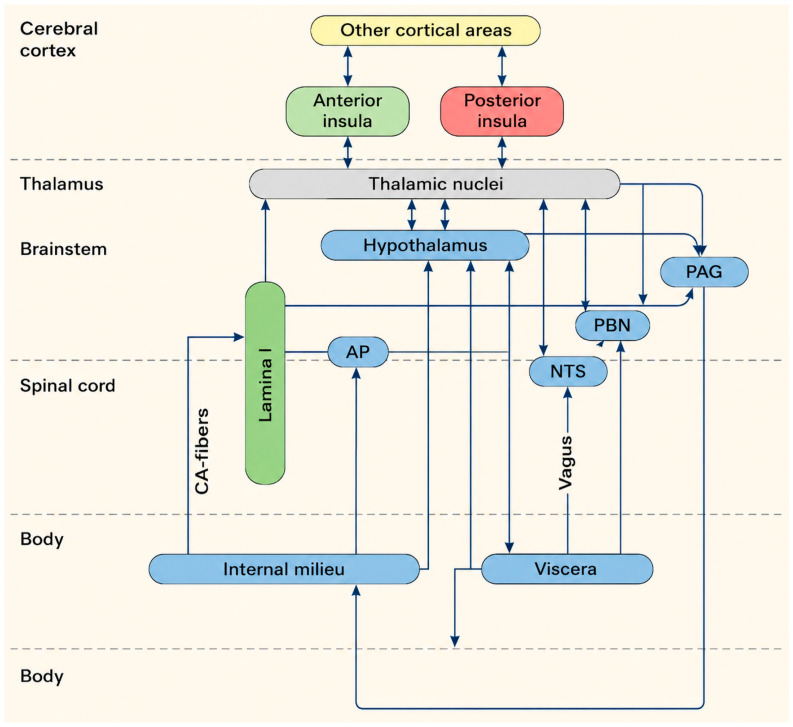
Interoceptive nuclei and ascending pathways involved in bodily-state representation. Interoceptive nuclei and ascending pathways contribute to the perception and representation of internal bodily states and may participate in emotional, autonomic, and regulatory responses associated with physiological deviation from homeostatic conditions.

From a neuroimmunology perspective, the relevance of the insula and associated salience networks (represented in Figure 4) lies not only in their role in interoception and self-referential processing but also in their potential vulnerability to developmental perturbation. The insula is positioned at the interface of autonomic regulation, visceral signaling, affective processing, and cognitive control. This anatomical and functional positioning raises the possibility that insula-centered circuits may be sensitive to biological processes that alter communication between peripheral physiological states and central neural systems.

Several features support this interpretation. First, the insula follows a prolonged and heterogeneous developmental trajectory, with anterior and posterior regions maturing at different rates. The anterior insula, in particular, undergoes extended postnatal development and remains plastic during early childhood. This extended developmental window may increase susceptibility to prenatal and early postnatal perturbations, including immune-related influences.

Second, the insula integrates interoceptive, autonomic, and affective signals, placing it at a point of convergence between systemic physiological states and central neural processing. Circulating cytokines, inflammatory mediators, autonomic dysregulation, and peripheral immune activation may therefore influence insula-related function indirectly through immune-brain communication pathways.

Third, insular function depends on tightly coordinated neurovascular and metabolic regulation. Immune-related alterations in blood-brain barrier dynamics, endothelial function, or microvascular integrity may influence the development and function of circuits that require precise neurovascular coupling. These effects should be interpreted cautiously, however, as current evidence supports plausibility rather than definitive region-specific causality.

Consistent with this framework, research indicates that individuals with autism may exhibit atypical surface morphometry in the insula, particularly in regions associated with social and emotional processing. Increased insular surface area has been correlated with poorer social functioning, suggesting that structural alterations in this region may contribute to social impairments commonly observed in ASD [36]. In addition, the right cingulate cortex, closely related anatomically and functionally to the insula, shows accelerated age-related reductions in surface area in individuals with ASD compared with typically developing controls. These findings suggest altered maturation of insula-cingulate networks involved in social cognition and emotional regulation [36,37].

### 4.5. Right-Hemisphere Timing, Autoimmunity, and Developmental Interpretation

The interaction between delayed right-hemisphere maturation and immune-related processes may be relevant to ASD, particularly for domains involving social communication, affective interpretation, and embodied self-awareness. Studies examining pragmatic language abilities sensitive to right-hemisphere dysfunction have shown that autistic individuals perform significantly worse than non-autistic controls on tasks involving humor, inference, and indirect requests, consistent with delayed or altered maturation of right-hemisphere systems involved in communication [38].

Additional evidence comes from studies of cerebral metabolic maturation. Investigations of preschool children with ASD have identified transient periods of frontal hypoperfusion at ages three to four years, resembling patterns observed in much younger, typically developing children. By ages six to seven years, frontal perfusion patterns approach typical developmental patterns, consistent with delayed rather than permanently arrested maturation [39]. Such findings support a developmental timing interpretation, in which immune-related processes may interact with delayed or altered circuit maturation rather than producing fixed structural deficits.

Autoimmunity has also emerged as a significant area of interest in ASD research. Evidence of autoimmune phenomena in individuals with autism suggests that altered immune responses may contribute to ASD-relevant developmental or phenotypic variation in some subgroups [40]. Antibodies directed against fetal brain proteins have been identified in some mothers of children with ASD, indicating a potential link between maternal immune responses and neurodevelopmental outcomes in offspring [41,42].

Further studies have shown elevated levels of pro-inflammatory cytokines and brain-specific autoantibodies in children with ASD, supporting associations between neuroimmune dysregulation and symptom expression and severity [43]. A positive correlation has been reported between antibodies against myelin basic protein and antibodies to viral antigens, suggesting interactions between infection and autoimmune mechanisms. These findings have contributed to proposals of possible autoimmune-related ASD subtypes in which immune processes may play a prominent role in pathogenesis [44,45].

### 4.6. Network Integration and Developmental Implications

The findings reviewed in this section support the interpretation that insula-centered and salience-related systems may represent one important site of convergence between neurodevelopmental timing, sensory-interoceptive processing, and immune-related vulnerability. However, this should not be interpreted as evidence that ASD is primarily an insular disorder. Rather, the insula is best understood as a candidate hub within a distributed developmental network that includes anterior cingulate, prefrontal, limbic, sensory, autonomic, and subcortical systems.

This reframing is central to the cascade model advanced in this review. Immune-related mechanisms may influence neural development through interactions involving cytokine signaling, microglial activation, synaptic refinement, blood-brain barrier dynamics, and peripheral immune-brain communication. These mechanisms may then alter the maturation of circuits involved in interoception, salience detection, sensory integration, affect regulation, and social cognition. Within this framework, insula-related findings provide a useful network-level example of how immune and developmental processes may converge, but they do not exhaust the range of neural systems potentially affected in ASD.

This perspective complements and extends existing frameworks of ASD, including predictive processing accounts, social brain network models, and sensorimotor integration theories. Rather than replacing these approaches, the present model proposes that temporally structured motor activity and neuroimmune signaling may provide a developmental substrate influencing the organization of these higher-order processes. In this sense, disruptions in timing and immune regulation may contribute to upstream developmental constraints shaping multiple downstream cognitive and social domains.

These network-level vulnerabilities provide a functional context within which immune-related processes may exert developmentally significant effects. In particular, regions involved in interoception, salience detection, and multisensory integration may be especially sensitive to inflammatory signaling, microglial activation, and disruptions in barrier function during critical developmental windows. This perspective establishes a direct conceptual bridge between network-level susceptibility and the immune-mediated mechanisms considered in the following section.

## 5. Immune Mechanisms in Autism Spectrum Disorder

### 5.1. Evidence of Neuroimmune Dysregulation in ASD

A substantial body of evidence indicates that immune-related alterations are present in a subset of individuals with autism spectrum disorder (ASD), involving both innate and adaptive immune systems [4,40]. Reported findings include changes in cytokine profiles, immune cell activation states, and inflammatory signaling pathways observed across peripheral and central compartments. Importantly, across studies, these immune-related findings are not uniformly observed and should be interpreted as probabilistic contributors within a heterogeneous, multi-factorial developmental framework rather than as defining features of ASD.

Peripheral immune alterations include elevated pro-inflammatory cytokines and chemokines, as well as dysregulated T-cell and B-cell responses. These immune profiles have been associated with variability in behavioral presentation and symptom severity, suggesting that immune activity may contribute to phenotypic expression in specific subgroups rather than representing a uniform etiological mechanism [43]. Accordingly, neuroimmune dysregulation in ASD is best understood as heterogeneous and context-dependent, varying across individuals and developmental stages.

### 5.2. Maternal Immune Activation and Developmental Timing

Maternal immune activation (MIA) represents one of the most consistently studied links between immune processes and neurodevelopmental outcomes. Epidemiological and experimental evidence indicate that maternal infections, inflammatory states, and neuroimmune dysregulation during pregnancy are associated with altered neurodevelopment in offspring [10,11].

Animal models suggest that prenatal exposure to inflammatory cytokines may disrupt neuronal migration, synapse formation, and cortical organization. These effects are highly dependent on developmental timing, with perturbations during sensitive windows exerting long-lasting effects on excitatory-inhibitory balance and circuit maturation. Within this framework, MIA is conceptualized as an early developmental influence that may affect downstream immune signaling and neural development.

### 5.3. Neuroinflammation, Microglial Function, and Synaptic Development

Neuroinflammatory processes have been reported in postmortem and neuroimaging studies of individuals with ASD, including evidence of microglial and astrocytic activation in cortical and cerebellar regions [46]. Microglia play an important role in synaptic pruning, neuronal maturation, and circuit refinement during development, supporting links between immune signaling and neural circuit formation. Importantly, this proposed pathway is supported by convergent experimental and human evidence. In maternal immune activation models, cytokine-dependent signaling, particularly involving IL-6 and IL-17A, has been associated with altered cortical development and behavioral phenotypes to produce behavioral phenotypes relevant to ASD [11,47]. In parallel, postmortem and neuroimaging studies have reported altered glial activation and atypical connectivity patterns in ASD [46,48]. While these findings do not establish a single causal pathway, they provide empirical support for the plausibility of immune-mediated modulation of synaptic development and large-scale network organization. Recent functional genomic studies provide direct evidence that ASD-associated risk genes influence microglial endocytic processes and synaptic pruning, establishing a mechanistic link between genetic susceptibility and circuit-level development [49].

Alterations in microglial activation may involve both excessive activation and impaired resolution of inflammatory responses. Such dysregulation has been proposed to influence synaptic pruning and plasticity, providing a plausible mechanistic pathway for atypical connectivity patterns observed in ASD, although direct causal evidence in humans remains limited [3]. However, it is important to distinguish between evidence of association and direct mechanistic causality, as many findings derive from postmortem or animal model studies.

### 5.4. Barrier Dynamics and Immune-Brain Communication

The blood-brain barrier (BBB) plays a critical role in regulating interactions between peripheral immune activity and the central nervous system. Evidence suggests that BBB integrity may be altered in some individuals with ASD, potentially allowing peripheral immune mediators to influence neural function [50]. These processes are increasingly understood within the broader framework of neuroimmune interfaces, in which brain barrier systems actively regulate immune–neural communication rather than serving as passive boundaries [51].

Inflammatory signaling, oxidative stress, and endothelial dysfunction may contribute to increased BBB permeability. During development, when barrier systems are still maturing, such changes may increase exposure of neural tissue to circulating cytokines and immune mediators. These processes provide a plausible pathway linking systemic immune activity to central neurodevelopmental outcomes, although region-specific effects remain to be fully established.

### 5.5. Specific Peripheral Immune Pathways Influencing Neurodevelopment

Beyond generalized inflammation, emerging evidence highlights specific immune pathways that provide more direct mechanistic links between peripheral immune activity and neural development.

One such pathway involves the interleukin-37 (IL-37) and interleukin-38 (IL-38) axis, which functions as an endogenous regulator of inflammatory responses. These cytokines suppress innate immune activation and modulate microglial activity. Dysregulation of this regulatory system may result in prolonged or insufficiently resolved inflammatory signaling during critical developmental windows, potentially affecting synaptic pruning and circuit stabilization.

Mast cells represent an additional mechanism linking peripheral immune activity to the central nervous system. Through the release of mediators such as tryptase, histamine, and cytokines, mast cells can influence BBB integrity by altering endothelial tight junctions. This process facilitates the entry of peripheral immune signals into the brain, providing a pathway for systemic immune activation to affect developing neural circuits.

The gut-immune-brain axis provides a further mechanistic pathway. Gut-derived T helper 17 (Th17) cells, particularly through IL-17A signaling, have been shown to influence cortical development in experimental models [52]. These findings link microbiome composition, peripheral immune activation, and neurodevelopment, suggesting that immune-mediated effects on brain organization may originate outside the central nervous system.

**Figure 5:**
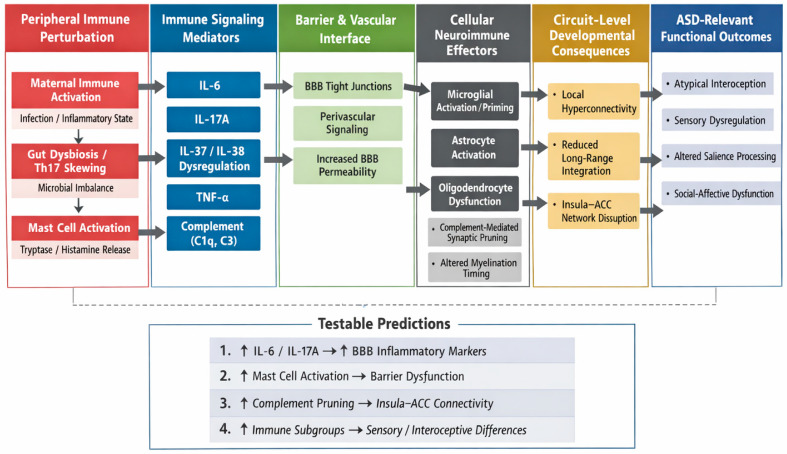
Multilevel neuroimmune cascade linking peripheral immune perturbation to neural circuit development in autism spectrum disorder. Schematic representation of a temporally structured developmental framework in which peripheral immune perturbations, including maternal immune activation, microbiome-associated immune signaling, and mast cell activation, may initiate molecular cascades involving pro-inflammatory cytokines (e.g., IL-6, IL-17A) and regulatory mediators (e.g., IL-37, IL-38). These signals interact with vascular and neuroimmune interfaces, potentially influencing blood–brain barrier dynamics and immune–brain communication during sensitive developmental periods. Within the central nervous system, these processes may modulate microglial activation, astrocytic signaling, complement-mediated synaptic pruning, neuronal migration, myelination, and excitatory–inhibitory balance. These cellular and molecular interactions may contribute to alterations in large-scale neural systems involved in interoception, salience processing, and sensory integration. The model generates testable predictions linking peripheral immune biomarkers, barrier dynamics, synaptic mechanisms, and network-level phenotypes, while emphasizing that these relationships are probabilistic, developmentally contingent, and heterogeneous across individuals.

These pathways refine the neuroimmune framework by specifying how distinct peripheral signals may differentially engage central developmental processes. These converging mechanisms motivate a systems-level integration in which peripheral immune signals, barrier dynamics, and central synaptic processes are organized into a unified developmental cascade (see Section 5.6 and Figure 5).

### 5.6. Mechanistic Integration Across Levels of Organization

We propose the following developmental neuroimmune cascade: early alterations in immune signaling influence microglial activation states, which in turn modulate synaptic pruning and stabilization during critical developmental windows. In this framework, early immune challenges, including maternal immune activation, microbiome-associated signaling, and peripheral inflammatory processes, initiate molecular pathways involving cytokines, regulatory immune mediators, and barrier dynamics. These processes influence central mechanisms such as microglial activation, astrocytic signaling, and complement-mediated synaptic pruning, which in turn affect neuronal migration, myelination, and excitatory-inhibitory balance. The cumulative effect of these processes is expressed at the level of large-scale neural systems, where alterations in connectivity and integration contribute to ASD-relevant phenotypes. This model emphasizes temporally structured interactions across biological scales and generates testable predictions linking immune biomarkers, developmental timing, and network-level outcomes (see Figure 5 for a schematic representation of this proposed multilevel cascade). This cascade formulation is explicitly mechanistic, linking peripheral immune perturbations to circuit-level outcomes through defined intermediate processes, including cytokine-mediated modulation of microglial activity, complement-dependent synaptic pruning, and alterations in excitatory–inhibitory balance.

Crucially, this framework does not assume linear causation but rather probabilistic interactions across levels of biological organization.

As illustrated in Figure 5, peripheral immune perturbations interact with barrier dynamics and central glial processes to influence synaptic refinement and large-scale network organization. This representation emphasizes multilevel coupling rather than a single causal pathway.

Within the central nervous system, these processes affect microglial activation, astrocytic signaling, and complement-mediated synaptic pruning, as well as neuronal migration, myelination, and excitatory-inhibitory balance. These cellular and molecular effects scale upward to influence large-scale neural systems. Circuits involved in interoception, salience processing, and sensory integration may be particularly sensitive due to their developmental timing and integrative roles, although such vulnerability should be interpreted within a distributed network framework.

This cascade model generates testable predictions linking immune biomarkers, barrier function, synaptic mechanisms, and network-level phenotypes, while acknowledging that these relationships are probabilistic and may vary across individuals.

### 5.7. Immune Heterogeneity and Clinical Interpretation

Despite substantial evidence for immune involvement, immune-related findings in ASD are not uniform. Considerable heterogeneity exists across individuals, reflecting differences in genetic background, environmental exposure, developmental timing, and methodological variation [13]. This variability complicates efforts to identify consistent biomarkers and highlights the importance of stratified approaches.

A mechanistically grounded biomarker framework requires specification of measurable variables across motor, neural, and immune domains. Candidate markers include variability in rhythmic motor output (e.g., gait periodicity and postural dynamics), neural indices of temporal coordination such as oscillatory coherence and phase-locking, and immune measures including cytokine profiles and markers of microglial activation. The predictive value of these markers is expected to lie in their temporal coupling rather than in isolated measures.

Current pharmacological treatments primarily target behavioral symptoms rather than underlying immune or neurodevelopmental mechanisms. Immune-targeted interventions remain limited by incomplete mechanistic understanding and variable efficacy, underscoring the need for cautious interpretation of translational implications. Accordingly, immune-related mechanisms are best interpreted as contributing to biologically defined subgroups within ASD rather than as universal features of the condition. Importantly, this framework generates testable predictions. Early disruptions in motor timing should precede and predict later impairments in social coordination and communication, and interventions targeting temporal motor coherence should produce measurable downstream effects on cognitive and social outcomes.

### 5.8. Summary and Link to Developmental Outcomes

Taken together, evidence from peripheral immune studies, neuroinflammatory findings, and developmental models indicates that immune processes may act as modulators of neurodevelopment rather than singular causal drivers. These findings provide the mechanistic basis for the neuroimmune dysregulation and developmental pathways elaborated in subsequent sections. Key immune-related findings in ASD are summarized in Table 2. The studies summarized below illustrate key empirical anchors for the proposed neuroimmune cascade, spanning peripheral immune signaling, central neuroinflammatory processes, and large-scale network-level alterations.

This framework generates specific, testable predictions. Early disruptions in motor timing and coordination should precede and predict later impairments in social communication, interoceptive integration, and large-scale network organization. Furthermore, interventions that enhance temporal motor coherence during early development should produce measurable downstream effects on cognitive and social outcomes. These predictions provide a basis for empirically evaluating the proposed neuroimmune cascade model and for distinguishing causal mechanisms from secondary effects in ASD.

We propose the following developmental cascade: early alterations in immune signaling influence microglial activation states, which in turn modulate synaptic pruning and stabilization processes during critical developmental windows. These changes bias the formation of neural connectivity patterns, particularly affecting the balance between local and long-range integration. Disruptions at this level constrain the emergence of coordinated motor timing and sensorimotor integration, which serve as foundational scaffolds for higher-order cognitive and social functions. Within this framework, behavioral and clinical manifestations of autism can be understood as downstream expressions of temporally misaligned neurodevelopmental processes, rather than isolated deficits. From this perspective, candidate biomarkers should reflect disruptions across multiple levels of this cascade.

## 6. Immune System Overview

### 6.1. Components of the Immune System

The components of the immune system are multifaceted and play a central role in maintaining physiological homeostasis and defending the organism against pathogenic challenges. In the context of autism spectrum disorder, understanding these components may help clarify potential mechanisms through which immune dysfunction may contribute to neurodevelopmental alterations [57].

The immune system is broadly divided into innate and adaptive immune responses. The innate immune system constitutes the first line of defense and includes physical barriers such as the skin and mucous membranes, as well as cellular components including natural killer (NK) cells, macrophages, and dendritic cells. NK cells, in particular, are potent effectors of immune homeostasis and are capable of recognizing and eliminating cells that display “non-self” or “missing-self” signals. These cells have been associated with a range of neurological and behavioral disorders, suggesting a potential association with ASD [58].

The adaptive immune system involves antigen-specific responses mediated primarily by lymphocytes, including T cells and B cells. T lymphocytes are further subdivided into functional subsets, such as helper T cells (Th) and cytotoxic T cells (Tc), each of which plays a distinct role in immune regulation and pathogen elimination. In individuals with ASD, evidence indicates increased activation of both Th1 and Th2 arms of the adaptive immune response, with a predominance of Th1-related activity. This imbalance may contribute to neuroinflammatory processes observed in ASD [43].

Cytokines, which function as signaling molecules produced by immune cells, are critical regulators of immune responses. Altered cytokine concentrations have been reported in peripheral blood, brain tissue, and cerebrospinal fluid of individuals with ASD, consistent with peripheral immune activation and possible downstream effects on central nervous system cytokine profiles via interactions with the blood-brain barrier [59]. Such alterations in the cytokine milieu may influence neuronal connectivity and synaptic function, thereby contributing to ASD pathophysiology [60].

Microglia, the resident macrophages of the central nervous system, represent another key immune component relevant to ASD. These cells play essential roles in synaptic pruning and neuronal development and have been implicated in developmental and neuroinflammatory processes of multiple neuropsychiatric conditions, including ASD [46]. Microglial activation and the associated release of pro-inflammatory cytokines may contribute to neuroinflammatory states, which are frequently reported in ASD.

In addition, the gut-brain axis, characterized by bidirectional communication between the gastrointestinal tract and the central nervous system, has received increasing attention. The gut microbiota exerts substantial influence on immune responses and has been associated with autoimmune and neurological disorders. Alterations in gut microbial composition have been documented in individuals with ASD, suggesting that microbial dysbiosis may contribute to neuroimmune dysregulation and neuroinflammation [33,61,62].

Collectively, the components of the immune system, including innate and adaptive immune cells, cytokines, microglia, and the gut microbiota, are closely involved in immune responses associated with ASD.

### 6.2. Immune System Function and Regulation

The immune system plays a critical role in maintaining internal homeostasis and protecting the body from pathogenic threats. It is composed of diverse cell populations and signaling molecules that operate in a coordinated manner to detect and eliminate foreign antigens. In the context of ASD, immune system dysfunction has been associated with neurodevelopmental variation related to the disorder.

Central to immune regulation is the cytokine network, which includes both pro-inflammatory and anti-inflammatory cytokines. Elevated concentrations of pro-inflammatory cytokines such as interleukin-6 (IL-6), tumor necrosis factor-alpha (TNF-α), and interleukin-1 beta (IL-1β) have been reported in individuals with ASD. These cytokines are released by immune cells in response to inflammatory stimuli and may interact with blood-brain barrier signaling and central immune pathways [43]. These findings are consistent with a possible role for systemic inflammation in the neurological and behavioral features of ASD.

Microglia also play a prominent role in immune regulation within the central nervous system. Upon activation, microglia secrete TNF-α and other inflammatory mediators that drive neuroimmune responses [63]. Sustained microglial activation may promote chronic neuroinflammatory states, potentially disrupting normal brain development and synaptic function.

Additional immune-related biomarkers, including brain-derived neurotrophic factor (BDNF) and S100B, have been examined in ASD. BDNF is involved in neural growth and synaptic plasticity, whereas S100B reflects aspects of neural immune activity. Abnormal levels of these markers have been associated with ASD, suggesting links between neuroimmune dysregulation and neurodevelopmental alterations [64].

The role of T lymphocytes, particularly CD4+ helper T cells and CD8+ cytotoxic T cells, has also been investigated in ASD. These cells are integral to adaptive immunity and influence immune responses through cytokine secretion and direct cell-cell interactions. Dysregulation of T-cell activity may therefore contribute to the immune abnormalities reported in ASD [40].

Regulatory mechanisms are essential for maintaining immune homeostasis. Transforming growth factor-beta 1 (TGF-β1) exerts strong downregulatory effects on T-cell and B-cell development and function and modulates the differentiation and activation of NK cells, dendritic cells, and macrophages. The distribution of TGF-β1 within the developing nervous system is consistent with a potential role in neuroimmune interactions [65].

Interactions between the gut microbiota and the immune system also contribute to immune regulation in ASD. Gut microbial composition influences systemic inflammation and neuroimmune signaling. Probiotic interventions have been explored as potential strategies to modulate these interactions and mitigate neuroimmune dysregulation associated with ASD [66].

### 6.3. Immune System and Brain Interaction

Interactions between the immune system and the brain represent an important area of investigation in understanding ASD pathophysiology. The gut-brain axis constitutes a bidirectional communication network linking the central nervous system and the enteric nervous system, thereby integrating cognitive and emotional processes with peripheral gastrointestinal function. The integrity of the blood-brain barrier and the gut-blood barrier is central to this interaction, and alterations in both barriers have been reported to be compromised in individuals with ASD.

Immune abnormalities are closely associated with ASD symptomatology. Elevated concentrations of pro-inflammatory cytokines, including IL-1β, IL-6, IL-12p70, and TNF-α, have been documented in individuals with ASD and have been shown to correlate with the severity of social impairments, suggesting possible associations between neuroimmune dysregulation and behavioral expression [43]. During fetal development, the maternal immune system is highly active and interacts with fetal immune cells, potentially influencing immune programming in the developing fetus [67]. This interaction is mediated in part by neonatal Fc receptors, which regulate the transplacental transfer of maternal IgG antibodies.

Microglia are central mediators of immune-brain interactions. Activation of microglia in response to environmental stressors and immune challenges may contribute to altered synaptic pruning and altered neuronal connectivity. Such changes may contribute to dysregulated fear responses and exaggerated autonomic reactions observed in ASD [46]. Additionally, CD4+ T cells and their cytokine product IL-17A are upregulated in several autoimmune and neuroinflammatory conditions, including ASD, further supporting investigation of immune pathways in ASD [11].

The enteric nervous system functions as a peripheral extension of the limbic system within the gastrointestinal tract, where it is exposed to mechanical, biochemical, and microbial influences. These influences can modulate gut-brain axis signaling and affect central nervous system function [68]. Immune abnormalities within this system may influence neurodevelopmental processes associated with ASD.

In summary, immune-brain interactions involve complex and dynamic processes that contribute to ASD development and progression.

## 7. Neuroinflammatory and Autoimmune Pathways in ASD

### 7.1. Evolution of Neuroimmune Models in ASD

Early models of autism spectrum disorder emphasized genetic and neurodevelopmental factors as primary contributors to ASD. Over time, immune abnormalities have become increasingly relevant to ASD research, particularly as studies began reporting altered cytokine profiles, immune-cell activity, gut-immune interactions, and autoantibody findings in subsets of individuals with ASD. Initial observations included increased pro-inflammatory cytokines such as IL-6 and TNF-α, together with decreased anti-inflammatory cytokines such as IL-10, suggesting a pattern of immune imbalance that may be associated with neurological and behavioral features of ASD.

Subsequent work expanded this view by identifying altered natural killer (NK) cell activity, maternal immune activation, gestational cytokine exposure, brain-reactive autoantibodies, and gut microbiota-related inflammatory pathways as potentially relevant to ASD-associated neurodevelopmental risk [69,70,71]. These findings do not support a single immune cause of ASD. Rather, they suggest that neuroimmune dysregulation may influence neurodevelopmental trajectories in biologically meaningful subgroups.

Across this evolving literature, a consistent theme is the convergence of genetic susceptibility, environmental exposure, cytokine imbalance, gut-immune signaling, and neurodevelopmental timing. This convergence is consistent with a developmental neuroimmune model in which immune processes may modify neural maturation rather than operate as isolated pathological events [72].

### 7.2. Cytokine Imbalance, Immune-Cell Function, and Developmental Risk

Current evidence implicates several interacting immune pathways in ASD, including altered cytokine profiles, immune-cell functional differences, gut dysbiosis, and maternal immune activation. Increased levels of pro-inflammatory cytokines such as IL-1β, IL-6, and TNF-α have been reported in children with ASD, and these mediators are biologically capable of affecting neuronal activity, synaptic function, and neurodevelopmental processes. Maternal cytokine activity during gestation is particularly relevant, potentially reflecting broader immune regulatory abnormalities.

Altered immune-cell function has also been reported. NK cells have been described as showing reduced cytotoxic activity or features of functional exhaustion in ASD, potentially reflecting broader immune regulatory abnormalities [73,74,75,76]. T-cell alterations, including changes in Th1/Th2 balance, Treg activity, and IL-17-related pathways, further suggest that both innate and adaptive immune systems may contribute to ASD-associated immune heterogeneity [77,78,79]. The role of IL-17-producing cells is particularly important, as IL-17 signaling has been associated with both protective and pathogenic effects depending on developmental context and immune regulation [80].

These immune findings must be interpreted within the broader heterogeneity of ASD. Immune abnormalities are not uniform across individuals and may vary according to developmental stage, comorbid medical features, environmental exposure, and genetic background. Thus, immunity is best understood as one component of a stratified neurodevelopmental model rather than as a universal mechanism.

### 7.3. Gut-Immune-Brain Signaling and Systemic Regulation

The gut-immune-brain axis represents a major pathway through which peripheral immune states may influence neurodevelopment. Recent studies have reported gut dysbiosis and inflammatory pathway enrichment in autistic children, including pathways involving IL-2, IL-6, IL-12 production, and Toll-like receptor signaling [81]. Alterations in gut microbiota may influence immune regulation, intestinal permeability, cytokine production, and systemic inflammatory tone, thereby potentially shaping neuroimmune communication. The gut–brain axis provides a framework through which microbial composition may influence immune signaling, neural circuitry, and behavior via bidirectional communication between the gastrointestinal, immune, and central nervous systems [82].

Dysbiosis may increase intestinal permeability, permitting microbial products and metabolites to enter circulation and trigger systemic immune activation. These processes may interact with neuroinflammatory pathways, microglial activation, and barrier function in ways relevant to ASD [83]. The gut microbiome may also interact with prenatal immune conditions and maternal inflammatory states, linking early environmental exposures to later neurodevelopmental outcomes.

Circadian disruption, metabolic regulation, and autonomic balance may further influence immune function. Circadian rhythms regulate immune-cell activity and inflammatory responsiveness, and disruption of these rhythms may amplify immune abnormalities relevant to neurodevelopment [84]. are consistent with a systems-level view in which gut, immune, metabolic, and neural processes interact across development.

### 7.4. Neuroinflammation and Glial-Mediated Developmental Effects

Inflammation has been widely discussed in relation to ASD pathophysiology. Elevated inflammatory markers, including IL-6, IL-17, TNF-α, IL-1β, RANTES, IL-8, and IFN-γ, have been reported in peripheral samples and immune-cell stimulation paradigms, although the cellular sources and developmental significance of these markers are not always clear [85,86,87]. In addition, oxidative stress interacts with inflammatory signaling pathways and may further disrupt neuronal function and synaptic integrity in ASD [88].

**Figure 6:**
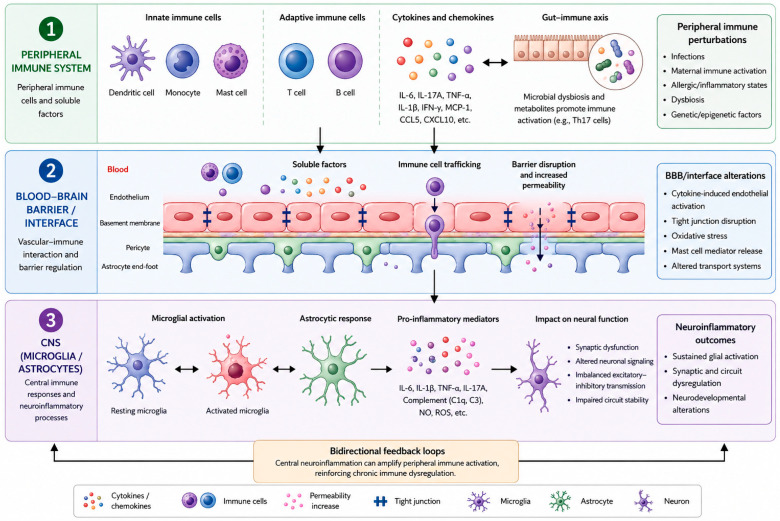
Interaction of peripheral and central immune processes associated with neuroinflammatory states in autism spectrum disorder. Schematic representation of bidirectional signaling between peripheral immune components, including cytokines, T cells, B cells, and gut-derived immune signals, and central nervous system processes. Elevated pro-inflammatory cytokines (e.g., IL-6, TNF-α, IL-1β) may influence microglial and astrocytic activation, potentially contributing to neuroinflammatory states within the brain. These central immune responses interact with synaptic function, neuronal signaling, and circuit stability, forming feedback loops that may reinforce inflammatory signaling across peripheral and central compartments. The model emphasizes that these interactions are dynamic, heterogeneous, and context-dependent rather than reflecting a single causal pathway.

Within the central nervous system, neuroinflammation has been associated with microglial and astrocytic activation, cytokine signaling, and chemokine activity [89]. Microglia and astrocytes regulate synaptic pruning, neuronal support, metabolic homeostasis, and inflammatory responses. Consequently, immune-mediated glial dysfunction may influence synaptic development, circuit refinement, and network maturation. The relationship among systemic inflammation, glial activation, and neural development is summarized schematically in Figure 6.

Cytokines such as IFN-γ and TNF-α may contribute to microglial and astrocytic activation, while adaptive immune abnormalities, including B-cell activation, autoantibody production, and dysregulated T-cell activity, may reinforce inflammatory signaling. These processes may form feedback loops linking peripheral immune activation with central neuroinflammatory states. However, the strength of evidence varies across models, and direct causal interpretation in human ASD remains limited.

### 7.5. Markers and Pathways of Neuroinflammation

Several markers and pathways have been proposed as relevant to neuroinflammation in ASD. Cytokine imbalance remains one of the most frequently reported findings, with dysregulation between pro-inflammatory and anti-inflammatory signaling described across multiple studies [90]. IL-17 signaling has received particular attention because maternal immune activation models associate IL-17A-dependent mechanisms with altered cortical development and autism-like behavioral outcomes [91].

Inflammasome activation has also been discussed as a pathway through which innate immune signaling may promote pro-inflammatory cytokine production and heightened inflammatory responsiveness [92]. Maternal infection, prenatal inflammation, immune-cell activation states, and environmental exposures during early development may further shape neuroinflammatory vulnerability [52,93,94,95].

A lifespan perspective is important because immune-neural interactions are not confined to prenatal development. Early childhood, adolescence, and adulthood may each involve different immune profiles [93] and different relationships between inflammatory signaling and neural function [47]. This developmental variability highlights the need for longitudinal models and biomarker strategies that distinguish state-dependent immune activity from stable developmental risk.

### 7.6. Autoimmunity and Autoantibodies in ASD

Autoimmune mechanisms have been increasingly implicated in ASD, particularly in subgroups with evidence of altered cytokine production, brain-reactive antibodies, gastrointestinal symptoms, or familial immune vulnerability. Immune involvement includes both innate and adaptive responses, with contributions from monocytes, neutrophils, CD4+ T cells, NK cells, B cells, and cytokine pathways such as IL-12 and IFN-γ [50].

Genetic studies have emphasized immune-related pathways and single nucleotide polymorphisms that may affect immune signaling during fetal development and in adult cortex [6]. Prenatal immune activation provides a further autoimmune-relevant pathway, as maternal cytokines including IL-6, IL-1β, and IL-17 have been linked to placental changes, fetal neurodevelopmental alteration, and later behavioral outcomes [96]. Gut-derived immune triggers, including bacterial components and microbial metabolites, may also initiate inflammatory cascades with central nervous system effects [97,98]. Contemporary models further demonstrate that maternal inflammatory signaling can alter fetal neurodevelopment through cytokine-dependent mechanisms, including IL-6 and IL-17 pathways that influence cortical organization and behavioral outcomes [99].

Autoantibodies against brain proteins have been reported in ASD and may interfere with neural development or function in vulnerable subgroups [100,101]. Autoantibody production is not restricted to the central nervous system; peripheral B-cell and T-cell alterations may contribute to broader systemic neuroimmune dysregulation and antibody generation [102]. Dysbiosis and increased intestinal permeability may further promote antigen translocation and autoimmune responses.

These findings support the possibility of immune-associated ASD subgroups, including proposed autoimmune-related phenotypes. However, the evidence should be interpreted cautiously. Autoantibody findings do not imply that ASD as a whole is autoimmune in origin; rather, they suggest that autoimmune processes may contribute to neurodevelopmental or behavioral variability in some individuals. Immune-modulating interventions have been explored, but their clinical use remains investigational and requires careful subgroup definition [103,104].

### 7.7. Genetic Susceptibility, Environmental Exposure, and Immune Stratification

Genetic predisposition to neuroimmune dysregulation may shape vulnerability to neuroinflammatory and autoimmune processes in ASD. Polymorphisms in immune-related genes, including genes encoding cytokines and cytokine receptors, may influence inflammatory responsiveness and neuroimmune signaling [105]. These genetic factors may interact with environmental exposures such as infection, toxicants, gut dysbiosis, and maternal inflammatory states.

Persistent immune activation, NK-cell exhaustion, elevated cytokines such as IL-6 and TNF-α, and microbiome-linked neuroimmune dysregulation may therefore reflect interactions between genetic susceptibility and environmental context rather than isolated abnormalities [106]. This framing is consistent with ASD heterogeneity and supports consideration of stratified approaches that integrate immune biomarkers, genetic profiles, microbiome measures, and developmental timing.

Dietary strategies, probiotic approaches, and other microbiota-directed interventions have been proposed as potential ways to modulate immune function, but evidence remains variable and should not be generalized across ASD populations [2,107]. Future studies should clarify which immune profiles are clinically meaningful, which are state dependent, and which may predict treatment responsiveness.

### 7.8. Section Summary

Taken together, neuroinflammatory and autoimmune pathways in ASD involve cytokine imbalance, altered immune-cell function, gut-immune-brain signaling, microglial and astrocytic activation, inflammasome activity, autoantibodies, and gene-environment interactions. These mechanisms do not support a single immune etiology of ASD. Instead, they reinforce the developmental cascade model in which immune processes interact with genetic susceptibility, environmental exposure, barrier function, and neural maturation to influence circuit-level and network-level outcomes.

## 8. Cytokines and Immune-Cell Effectors in ASD

### 8.1. Cytokine Profiles and Immune Imbalance

Cytokine dysregulation represents one of the most frequently reported immune findings in autism spectrum disorder (ASD). Pro-inflammatory cytokines, including interleukin-1β (IL-1β), interleukin-6 (IL-6), and tumor necrosis factor-α (TNF-α), have repeatedly been reported at elevated levels in subsets of individuals with ASD. These mediators are biologically relevant because they may influence neuronal differentiation, synaptic function, microglial activation, and blood-brain barrier signaling. However, their presence should not be interpreted as evidence of a uniform inflammatory state across all individuals with ASD, but rather as a marker of immune heterogeneity within biologically defined subgroups [108,109,110].

Maternal immune activation (MIA) provides an important developmental context for interpreting cytokine findings. Prenatal immune challenge may elevate cytokines, particularly IL-6, and has been associated in experimental and epidemiological studies with altered neurodevelopmental outcomes in offspring [10,11,53]. Gut microbiota differences in ASD may also influence cytokine production and systemic immune tone, reinforcing the relevance of gut-brain-immune interactions [101]. These findings are consistent with a model in which cytokines act as developmentally timed modulators of neural maturation rather than as isolated disease markers.

Anti-inflammatory cytokines are equally important for interpreting ASD-related immune imbalance. Interleukin-10 (IL-10) and transforming growth factor-beta (TGF-β) regulate immune activation, promote tolerance, and help constrain inflammatory responses. Altered IL-10 and TGF-β signaling has been reported in ASD, suggesting impaired regulation of immune activation in some individuals [65,111,112]. The balance between pro- and anti-inflammatory signaling may therefore be more informative than any single cytokine measure.

Cytokine imbalance has also been associated with cognitive, behavioral, and medical features in ASD. Elevated cytokine levels have been inversely correlated with intellectual functioning in some autistic children, particularly among those with sleep disturbances and aggressive behaviors, and increased expression of IL-1β, IL-6, IL-10, and MCP-1 has been reported in these populations [43]. ASD-asthma comorbidity has also been associated with elevated IL-17A, suggesting overlap between immune and neurodevelopmental phenotypes [113]. These associations support the use of stratification approaches, including bioinformatics and machine learning methods, to identify immune-defined ASD subgroups [95,114,115]. Recent multi-biosample analyses further suggest relationships between cytokine profiles in ASD and gut microbial composition, supporting an integrated immune–microbiome contribution to neurodevelopmental outcomes [116].

### 8.2. Adaptive Immune Cells: T Cells and B Cells

T cells and B cells represent major adaptive immune contributors to ASD-related immune heterogeneity. T helper 17 (Th17) cells are particularly relevant because they differentiate in the presence of TGF-β and IL-6 and are involved in inflammatory and autoimmune responses [117]. Elevated IL-17A, produced by Th17 cells, has been associated with ASD symptom severity, while increased mucosal IL-17 production accompanied by reduced regulatory IL-10 suggests disrupted mucosal immunity and intestinal barrier regulation [118,119].

B cells have also been implicated through altered frequency, function, and antibody production. B-cell-mediated mechanisms may contribute to immune-neural interactions through autoantibody generation and inflammatory signaling, particularly in ASD subgroups with evidence of autoimmune features [120]. Functional polymorphisms in macrophage inhibitory factor (MIF), which affects both innate and adaptive immunity, have also been associated with ASD severity [121]. Together, these findings indicate that adaptive immune abnormalities may contribute to ASD phenotypic variability through cytokine signaling, mucosal immune disruption, and autoantibody-related pathways [43].

### 8.3. Central Immune Effectors: Microglia and Astrocytes

Microglia and astrocytes are key cellular mediators linking immune activation to neural development. Microglia participate in synaptic pruning, respond to inflammatory cues, and participate in circuit refinement. Astrocytes contribute to neurotransmission, blood-brain barrier integrity, metabolic support, and synaptic modulation. In ASD, microglial activation has been frequently reported and is associated with neuroinflammatory processes [46].

Astrocytic dysfunction may further amplify immune-neural dysregulation, particularly when microglia-astrocyte interactions become altered. Autoantibodies targeting glial fibrillary acidic protein (GFAP) and neuro-axon filament proteins have been reported, suggesting possible autoimmune targeting of glial elements in some ASD subgroups [122]. Gut microbiota-related influences on glial activity through inflammatory mediators such as IL-1β provide another pathway linking peripheral immune states to central neuroimmune function [123]. Maternal immune activation models further demonstrate that IL-17A-producing T cells may influence glial activation and offspring neurodevelopment [11].

### 8.4. Innate Immune Cells and Regulatory Function

Natural killer (NK) cells and other innate immune populations contribute to ASD-related immune heterogeneity. Alterations in NK cell frequency, cytotoxicity, and gene expression have been reported, suggesting dysregulated innate immune responses [124]. Cytokines such as IL-6 and TNF-α can modulate NK cell function, and chronic exposure to elevated inflammatory signals may impair NK cell efficacy [43]. NK-cell interactions with dendritic cells, macrophages, and T cells may further influence immune coordination, inflammatory signaling, and neuroimmune communication [125].

Regulatory immune mechanisms are equally important. Reduced regulatory T-cell function, including reduced CD4^+^CD25high Tregs and altered FOXP3 expression, may impair immune suppression and contribute to chronic inflammatory persistence [126]. Microglial abnormalities in ASD have also been described in relation to altered density, morphology, autophagy, and disrupted interactions with astrocytes and oligodendrocytes, suggesting relationships between immune activation and synaptic and cellular homeostasis [63]. Gut-associated markers such as S100B have been proposed as potential indicators of astrocytic or enteric glial involvement [127].

### 8.5. Immune-Cell Communication and Stratification

Immune-cell communication occurs through cytokines, chemokines, and pattern-recognition pathways. Elevated IFN-γ and TNF-α may influence microglial and astrocytic activation, while B-cell activation and autoantibody production may contribute to maladaptive immune-neural signaling [46,128]. Innate immune pathways, including Toll-like receptor and NOD signaling, may further modulate inflammatory responses relevant to ASD [129]. Gut microbiota interactions with immune cells may contribute to systemic inflammation with downstream CNS effects, reinforcing gut-brain-immune coupling [56].

Sex hormones may also shape immune-cell activity. Estrogens can enhance humoral immunity, whereas androgens often exert immunosuppressive effects, potentially contributing to sex differences in immune profiles and ASD presentation [130]. Comorbidities such as epilepsy, gastrointestinal dysfunction, asthma, and sleep disturbance may be associated with distinct cytokine or immune-cell profiles, further supporting consideration of stratified immune phenotyping [65,131,132,133,134].

Overall, cytokines and immune-cell effectors should be understood as interacting components of a broader developmental neuroimmune cascade. Their relevance lies not in any single marker or cell type, but in the coordinated patterns through which immune signaling, glial function, barrier integrity, microbiome activity, sex-linked immune modulation, and developmental timing may converge to influence neural circuit maturation and ASD-related heterogeneity.

## 9. Genetic and Environmental Modifiers of Neuroimmune Risk

### 9.1. Genetic Susceptibility and Immune-Neurodevelopmental Vulnerability

The genetic basis of autism spectrum disorder (ASD) is complex and involves multiple genes, variants, and regulatory mechanisms that interact with environmental influences across development. Mutations associated with neurodevelopmental disorders (NDDs) may exert subtle but consequential effects on differentiation-related processes rather than producing direct cellular toxicity. This pattern suggests that genetic contributions to ASD may influence the timing, coordination, and stability of central nervous system (CNS) development.

Genetic susceptibility may also shape immune response patterns relevant to ASD. Immune abnormalities, including T-cell dysregulation, altered cytokine levels, elevated IL-17, Th17/Treg imbalance, macrophage polarization changes, and altered TNF-α signaling, have been discussed in relation to ASD risk and severity [2,14,71]. These findings suggest that genetic variation may influence both neural development and immune regulation, thereby potentially shaping individual vulnerability to neuroimmune perturbation.

Autoimmune-related mechanisms further support the relevance of genetic susceptibility. Autoimmunity, autoantibody involvement, and family histories of autoimmune disorders have been discussed in ASD, suggesting that immune maladaptation may contribute to developmental vulnerability in specific subgroups [135]. However, these findings should not be interpreted as evidence that ASD is primarily autoimmune in origin. Rather, they support a stratified model in which genetic predisposition may increase susceptibility to immune-related developmental effects.

A reductionist approach that treats ASD as a set of isolated biological abnormalities risks obscuring the interacting networks through which genetic, immune, environmental, and developmental factors shape phenotype [1]. Within the framework advanced here, genetic susceptibility is therefore understood as one component of a broader developmental cascade, influencing how neural systems may respond to immune signaling, environmental exposures, and early-life physiological conditions.

### 9.2. Genetic Variants, Epigenetic Regulation, and Developmental Timing

Genetic mutations and variants implicated in ASD include single-nucleotide polymorphisms (SNPs), copy number variations (CNVs), and mutations in genes involved in neurodevelopment and immune function. Some variants may influence stress responsivity, hypothalamic-pituitary-adrenal (HPA) axis activity, and behavioral regulation, as illustrated by the discussion of the N363S SNP in relation to comorbid neurodevelopmental and behavioral conditions [136]. These findings are consistent with evidence that ASD-associated genetic variation may converge on intracellular signaling pathways that regulate both neural development and immune function, including cytokine-mediated and inflammatory signaling cascades [137].

Epigenetic mechanisms provide an additional layer through which genetic susceptibility and environmental exposure may interact. Alterations in the serotonin transporter gene (SLC6A4), including methylation changes associated with clinical features and cortical thickness variation, suggest overlap among genetic, epigenetic, and neurodevelopmental pathways across ASD and related conditions [8]. Similarly, down-regulated micro-RNAs targeting circadian rhythm pathways further support gene regulation and biological timing in ASD heterogeneity [72].

Maternal immune activation (MIA) represents a major context in which genetic susceptibility and prenatal environment converge. Offspring exposed to MIA may exhibit neurodevelopmental abnormalities and primed immune responses, potentially mediated by maternal cytokines such as IL-17A [2]. MIA-related alterations in maternal gut bacteria may also contribute to sustained immunological changes in offspring, including effects demonstrated in fecal transfer experiments in germ-free mice [137]. These findings support the view that genetic risk is expressed within developmental environments rather than operating independently.

The gut microbiome may also participate in epigenetic regulation. Early colonization patterns, mode of delivery, antibiotic exposure, and microbial metabolites may influence immune signaling and gene expression. Microbial products such as folate and butyrate have been discussed as relevant to epigenetic regulation and potential microbiome-mediated modulation of ASD-related symptoms [1,138]. Altered brain-derived neurotrophic factor (BDNF), which supports neuronal growth and synaptic plasticity, further supports the convergence of genetic, immune, and developmental pathways in ASD [71].

### 9.3. Gene-Environment Interactions and Barrier Function

Gene-environment interactions are central to the developmental neuroimmune cascade proposed in this review. Environmental exposures may influence gene expression, immune regulation, barrier integrity, and neural maturation, while genetic susceptibility may influence the degree of vulnerability to those exposures.

Alterations in blood-brain barrier function and intestinal tight junction proteins have been reported in ASD and linked to dysbiosis and altered bacterial metabolites [12]. This provides a plausible biological interface through which genetic susceptibility, gut ecology, immune activation, and neural development may interact. Altered barrier function may amplify systemic immune signaling and facilitate communication between peripheral inflammation and central neurodevelopmental processes.

Prenatal stress represents another example of environmental influence interacting with biological susceptibility. Intrauterine inflammation, serotonergic dysfunction, and microbe- or CCL2-dependent mechanisms have been associated with long-term behavioral outcomes [6]. Early-life events, including first-trimester stressors, gestational age variation, and obstetric complications, may similarly influence gene expression and developmental trajectories [8].

Other environmental exposures have also been discussed in relation to ASD and related neurodevelopmental conditions. Low-level, low-frequency electromagnetic radiation has been proposed as a factor that may influence blood-brain barrier permeability and contribute to sleep, memory, and concentration disturbances [139]. Such claims require cautious interpretation, but they illustrate the broader principle that environmental influences may interact with susceptibility factors through barrier, immune, and neurophysiological pathways.

Circadian gene variants and environmental modulators such as light exposure, social cues, and daily schedules provide another example of gene-environment interaction. Circadian disruption, shaped by both genetic and environmental inputs, may influence cognition, behavior, immune regulation, and developmental timing [72]. Regulatory considerations involving the mother, fetus, and neonate further emphasize the importance of prenatal and early-life exposure windows in shaping neurodevelopmental outcomes [1].

### 9.4. Section Summary

Genetic and environmental factors should not be treated as separate explanatory domains in ASD. Rather, they interact across developmental time to shape immune regulation, barrier function, microbiome composition, epigenetic control, stress responsivity, and neural circuit maturation. Within the developmental neuroimmune cascade model, genetic susceptibility contributes to vulnerability, while environmental exposures influence when, where, and how that vulnerability is expressed. This framework supports stratified approaches to ASD research that integrate genetics, immune profiling, microbiome measures, and developmental timing without reducing ASD to any single causal pathway.

## 10. Environmental Modulators of Neuroimmune Development

### 10.1. Prenatal and Perinatal Immune-Related Exposures

Prenatal and perinatal environments represent critical windows during which environmental exposures may influence neurodevelopmental trajectories in ASD. Among these, maternal immune activation (MIA) remains one of the most consistently studied risk factors. Maternal infection and inflammatory states during pregnancy can elevate cytokines such as IL-6, IL-1β, and IL-17, which may cross the placental interface or alter placental signaling, thereby potentially influencing fetal brain development [11,13,115]. These effects appear to be highly dependent on developmental timing, with specific gestational windows conferring differential vulnerability.

Maternal immune-related conditions, including asthma and allergic disease, have also been associated with increased ASD risk, particularly when active during mid-gestation [2,13]. These conditions involve cytokine release by mast cells and dendritic cells, including IL-1β, IL-4, IL-5, IL-6, IL-12, IL-13, IFN-γ, and TNF-α, which may influence fetal immune programming and neurodevelopment. However, these associations should be interpreted cautiously, as they likely reflect complex interactions among maternal physiology, immune signaling, and fetal susceptibility rather than direct causal pathways.

Circadian disruption during pregnancy has also been discussed as a potential modifier of neurodevelopmental risk. Disruption of circadian rhythms may affect immune regulation, hormonal signaling, and metabolic processes, and animal models suggest that prenatal circadian disturbance may influence immune function and developmental outcomes [79,84].

Perinatal factors, including prematurity and early-life infections, have also been associated with increased neurodevelopmental risk [94]. These exposures may reflect underlying vulnerability in immune regulation, barrier function, or developmental timing. Sex-dependent effects, such as differential outcomes associated with perinatal interventions, further highlight the importance of developmental context and biological variability [8].

Taken together, prenatal and perinatal exposures are best understood as modulators of developmental neuroimmune processes, influencing susceptibility rather than determining outcome.

### 10.2. Environmental Toxicants and Neuroimmune Disruption

Environmental toxicants have been investigated as potential contributors to ASD risk through their effects on neurodevelopment and immune regulation. Heavy metals such as lead, mercury, and cadmium may influence blood-brain barrier dynamics and accumulate in neural tissue, where they may interfere with neuronal development, synaptic signaling, and metabolic processes [136].

Pesticides, particularly organophosphates, have also been associated with neurodevelopmental risk. These compounds inhibit acetylcholinesterase and may disrupt neural signaling, with animal models demonstrating behavioral and developmental effects relevant to ASD [73,74,75]. Industrial chemicals such as polychlorinated biphenyls (PCBs) and bisphenol A (BPA) have been associated with endocrine disruption, altered thyroid hormone signaling, and neurodevelopmental effects [127,140].

Importantly, many toxicants also interact with immune function. Heavy metals, pesticides, and air pollutants have been described as potentially capable of activating microglia and promoting inflammatory signaling, potentially contributing to neuroinflammatory processes [84]. Prenatal exposure is particularly relevant, as the developing brain and immune system may be more vulnerable to environmental perturbation. Air pollution, which represents a complex mixture of toxicants, has also been associated with increased ASD risk, although causality remains difficult to establish due to confounding environmental and socioeconomic factors [70,140,141].

Overall, toxicant exposures are best conceptualized as environmental modifiers that may influence neurodevelopment through combined effects on neural, immune, and barrier-related processes.

### 10.3. Microbiome and Gut-Immune-Brain Interactions

The microbiome represents a major environmental interface linking immune regulation and neurodevelopment. The gut microbiota plays a central role in immune modulation, metabolic signaling, and maintenance of barrier integrity. Alterations in microbial composition have been reported in individuals with ASD and may influence cytokine production, immune-cell differentiation, and systemic inflammatory tone.

Intervention studies have explored whether modifying the microbiome may influence ASD-related symptoms. For example, administration of Bifidobacterium infantis with bovine colostrum has been associated with improvement in gastrointestinal symptoms and some behavioral measures in autistic children [142]. These effects have been associated with changes in cytokine profiles, including reductions in TNF-α and IL-13 [43,125]. While such findings are promising, they remain preliminary and require further replication and mechanistic clarification.

The gut-brain axis involves neural, endocrine, and immune pathways. Vagal signaling, hypothalamic-pituitary-adrenal (HPA) axis modulation, and cytokine-mediated communication all contribute to this bidirectional system [61]. Dysregulation of this axis has been proposed as a factor that may influence behavior, cognition, and affective processing in ASD.

Microglial activation may represent a key point of convergence for microbiome-related immune signaling. Gut-derived inflammatory signals may influence microglial activity, providing one pathway through which peripheral immune states may influence central nervous system development [46]. Additional processes, including oxidative stress, mitochondrial dysfunction, and altered calcium signaling, have also been associated with microbiome-related immune effects [73,75,133].

Genetic factors, including variation within the major histocompatibility complex, may further influence microbiome composition and immune responses, reinforcing the interaction between host genetics and environmental exposures [125]. Non-pharmacological approaches, including behavioral and physiological regulation strategies, have also been proposed as modulators of gut-brain-immune function, although evidence remains limited [1,132].

### 10.4. Section Summary

Environmental exposures relevant to ASD, including prenatal immune challenges, toxicant exposure, and microbiome-related factors, interact with genetic susceptibility and developmental timing to influence neuroimmune processes. These exposures do not act in isolation but may converge on shared biological pathways, including cytokine signaling, barrier function, glial activation, and metabolic regulation. Within the developmental cascade framework, environmental factors shape when and how underlying vulnerabilities are expressed, contributing to the heterogeneity observed across ASD populations.

## 11. Final Common Neurobiological Pathways: Glia, Barrier Function, and Neurotransmitter Balance

### 11.1. Microglia, Astrocytes, and Developmental Circuit Remodeling

Microglia are resident immune cells of the central nervous system and play essential roles in synaptic pruning, neurogenesis, and maintenance of neural homeostasis. In ASD, altered microglial activation has been implicated in neurodevelopmental disruption, particularly in the context of prenatal and early postnatal immune challenges. Evidence suggesting neuroimmune dysregulation at or near birth, maternal autoantibodies targeting fetal brain proteins, and later changes in brain structure and function support the possibility that early immune perturbations may influence developmental circuit remodeling in vulnerable subgroups [143]. These processes provide a plausible mechanistic bridge between immune signaling and large-scale network organization, as alterations in synaptic pruning and glial-mediated modulation of neural activity may influence the balance between local circuit refinement and long-range connectivity. At the systems level, these processes are likely to involve coordinated interactions among cortico-striatal, cerebellar, and thalamocortical circuits, which are involved in temporal prediction, sensorimotor integration, and large-scale network synchronization. Disruptions within these timing-sensitive systems may help link early immune perturbations to later differences in connectivity, coordination, and behavioral organization observed in ASD.

Elevated pro-inflammatory cytokines, including TNF-α, IL-6, IL-8, IL-1β, and IL-12p40, have been reported in ASD contexts and may influence microglial activation states [144,145]. Microglia also interact with neurotransmitter systems, including serotonin, dopamine, and glutamate, suggesting reciprocal relationships among immune signaling, neural activity, and synaptic regulation [146]. Immune responses to neural antigens, including myelin basic protein and anti-serotonin 5HT1A receptor autoantibodies, further support models in which autoimmunity, cytokine imbalance, and glial activity shape the broader neuroimmune environment [147,148].

Gut-derived inflammation, increased intestinal permeability, HMGB1 signaling, altered T- and B-cell profiles, and prenatal exposures such as saturated fatty acids may also prime microglial responses through systemic immune pathways [144,147]. Astrocytes participate in these processes by regulating synaptogenesis, myelination, metabolic support, and inflammatory signaling. Disturbances in microglia-astrocyte interactions may therefore amplify neuroinflammatory effects on synaptic function, neurogenesis, and myelination [145,146].

Overall, glial-mediated neuroinflammation should be understood as a convergence point where genetic, immunological, metabolic, gut-derived, and environmental influences may affect circuit refinement. These findings support interest in microglial activation and inflammatory signaling as potential targets for investigation, while avoiding the assumption that glial activation is uniform or causal across all ASD phenotypes.

### 11.2. Blood-Brain Barrier Dysfunction and Immune-Brain Access

The blood-brain barrier (BBB) is essential for maintaining central nervous system homeostasis by regulating the movement of molecules, immune mediators, and cells between the circulation and neural tissue. In ASD, BBB integrity has been discussed as potentially altered in some individuals, providing one pathway through which peripheral immune activity may influence central neurodevelopment. Neurovascular alterations further contribute to impaired barrier integrity, highlighting the role of cerebrovascular signaling in modulating immune access to the central nervous system [149].

Inflammatory cytokines, including IL-6, IL-1β, IL-8, and TNF-α, may affect endothelial function and tight-junction integrity, potentially altering BBB permeability and immune-brain communication [139,150,151]. Microglial activation may further interact with BBB function, creating feedback loops in which systemic inflammation, glial activation, and barrier dysfunction reinforce one another. MHC-I signaling has also been discussed in relation to excitatory-inhibitory balance, synaptic regulation, and homeostatic plasticity, linking immune-related molecules to circuit-level development [150,151].

Developmental timing is critical. The BBB matures across early life, and maternal inflammation, prenatal cytokine exposure, autoantibodies, and early-life immune challenges may affect later barrier vulnerability [145,152]. Gut inflammation and increased intestinal permeability may also contribute to systemic inflammatory signaling, potentially allowing microbial metabolites and immune mediators to influence BBB dynamics and neuroimmune activation [139].

Oxidative stress and mitochondrial dysfunction provide additional links between immune activation and barrier disruption. Mitochondrial impairment can increase oxidative stress, which may compromise endothelial cells and tight junctions, thereby linking metabolic abnormalities to BBB permeability [146]. S100B and other inflammatory mediators may further amplify cytokine signaling and contribute to BBB-related neuroimmune processes [139]. IGF-1 signaling, white matter development, myelination, and oligodendrocyte differentiation have also been discussed in relation to ASD-like phenotypes and learning challenges [146].

Thus, altered BBB dynamics are best conceptualized as part of a broader neuroimmune interface rather than as a standalone causal mechanism. Altered BBB dynamics may help explain how peripheral neuroimmune dysregulation, gut-derived inflammation, oxidative stress, and autoantibody-associated processes influence neural development in selected ASD subgroups.

### 11.3. Neurotransmitter Systems as Downstream Targets of Neuroimmune Signaling

Neurotransmitter imbalance represents a key downstream pathway through which neuroimmune dysregulation may influence neural function in ASD. Glutamate and GABA regulate excitatory and inhibitory signaling, and their balance is essential for synaptic stability, plasticity, and information processing. Altered glutamatergic signaling, including elevated glutamatergic compounds, has been reported in ASD and may contribute to hyperexcitability and altered connectivity [153,154]. Reduced GABAergic signaling has also been described and may contribute to sensory processing abnormalities, social impairment, and repetitive behaviors [155,156].

Immune signaling may affect excitatory-inhibitory balance through several mechanisms. Pro-inflammatory cytokines may influence glutamate release and uptake, alter receptor function, and affect GABAergic signaling, while oxidative stress may impair glutamate transport and intensify excitatory vulnerability [154,156]. These pathways may link neuroinflammation to circuit instability and provide a mechanistic bridge between immune activity and neural-network function.

Serotonin and dopamine systems are also relevant to ASD neurobiology. Serotonin contributes to mood regulation, social behavior, and cognition, while dopamine supports reward processing, motor function, and executive regulation. Alterations in serotonergic and dopaminergic signaling have been discussed in ASD, with clinical variability reflected in mixed responses to serotonin-targeting interventions [157,158,159]. Importantly, serotonergic and dopaminergic receptors are expressed on immune cells, supporting bidirectional interactions between neurotransmitter systems and cytokine production [160].

Neurotransmitter systems also interact with circadian regulation, sleep-wake cycles, metabolic processes, and immune function. Circadian disruption may therefore amplify both neurochemical and neuroimmune dysregulation, increasing symptom burden in some individuals. Therapeutic strategies targeting glutamate, GABA, serotonin, and dopamine pathways have been explored, but their relevance likely depends on subgroup biology and developmental timing rather than a single uniform ASD mechanism [161,162].

### 11.4. Section Summary

Microglial activation, astrocytic dysfunction, BBB permeability, oxidative stress, mitochondrial impairment, and neurotransmitter imbalance represent convergent pathways through which neuroimmune perturbations may influence ASD-relevant circuit development. These mechanisms connect peripheral immune signaling to synaptic pruning, myelination, excitatory-inhibitory balance, and large-scale network maturation. Their relevance is likely subgroup-specific and developmentally dependent, reinforcing the need for stratified, biomarker-informed approaches rather than generalized causal claims.

## 12. Discussion

A central aim of this work is to advance a developmental neuroimmune cascade model of autism spectrum disorder, integrating evidence across immunology, neurodevelopment, and systems neuroscience to propose a mechanistic framework linking early immune signaling to altered neural circuit formation. Rather than viewing neuroimmune dysregulation as a downstream correlate of neural dysfunction, we propose that immune signaling, particularly microglial activity and cytokine-mediated modulation, may participate in the timing and coordination of synaptic development, pruning, and large-scale network formation. Within this framework, ASD may be understood as a divergence in neurodevelopmental timing, in which alterations in immune-mediated processes may disrupt the coordinated emergence of functional connectivity. This perspective attempts to integrate previously disparate findings across molecular, cellular, and systems-level domains into a coherent developmental model, emphasizing timing as a critical and underappreciated dimension of neurobiological organization. Our framework proposes a developmentally structured model intended to move beyond purely descriptive heterogeneity toward a causally structured developmental model in which immune-neural interactions may function as important developmental constraints.

ASD is clinically and biologically heterogeneous, and immune-related findings are unlikely to apply uniformly across all individuals [1,2]. The evidence reviewed here supports a subgroup-relevant framework in which neuroimmune dysregulation may modify neurodevelopmental trajectories in specific biological contexts rather than represent a singular cause of ASD. This interpretation is consistent with the difficulty of assigning ASD pathophysiology to any single mechanism and with evidence implicating genetic, immune, environmental, metabolic, and neural systems in interacting developmental pathways [163,164,165].

A central feature of this framework is the interaction between peripheral immune activation and neural circuit development. Maternal or early-life immune activation may alter cytokine signaling, including IL-6 and IL-17A, with potential downstream effects on microglial activation, complement-mediated synaptic pruning, and circuit refinement [11,47,96]. These immune-mediated processes may influence neuronal migration, synaptic maturation, myelination, and excitatory-inhibitory balance during sensitive developmental windows [98,102,103,166].

At the systems level, disrupted synaptic refinement and altered myelination may contribute to large-scale connectivity differences reported in ASD, including changes in default mode, salience, fronto-temporal, and interoceptive networks [48,167,168,169]. Within this framework, insula-centered and cingulate-related systems are considered candidate sites of convergence because of their roles in interoception, salience processing, autonomic regulation, and affective-cognitive integration. However, these systems should be understood as part of a distributed network vulnerability model rather than as uniquely privileged sites of pathology.

**Figure 7:**
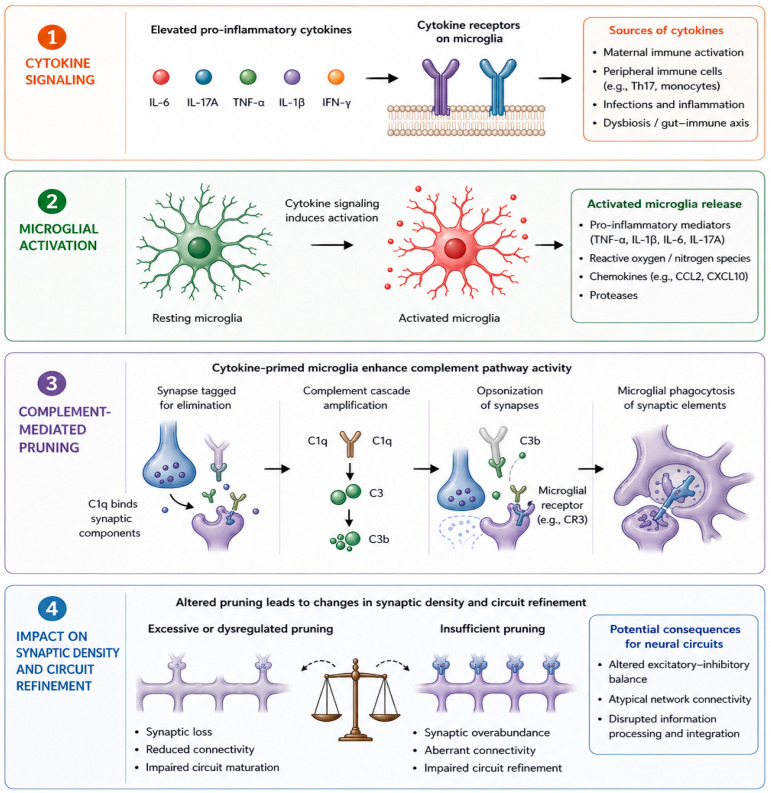
Neuroimmune pathways linking immune activation to altered neural connectivity in autism spectrum disorder. Simplified conceptual representation of selected neuroimmune mechanisms that may contribute to altered neural connectivity during development. Maternal immune activation during pregnancy may elevate inflammatory cytokines such as interleukin-6 (IL-6) and interleukin-17A (IL-17A), which may influence fetal brain development. These cytokine signals may modulate microglial activation within the developing central nervous system. Activated microglia participate in complement-mediated synaptic pruning processes involving components such as C1q and C3 that normally contribute to neural circuit refinement during development. Dysregulation of these processes may alter synaptic elimination and stabilization, potentially contributing to atypical synaptic density and altered patterns of neural connectivity. Such alterations in circuit maturation may contribute to behavioral phenotypes associated with autism spectrum disorder. This figure represents a reduced conceptual model of selected synaptic and microglial processes within the broader developmental neuroimmune cascade described in the text.

Immune signaling also interacts with metabolic and mitochondrial processes. Mitochondrial dysfunction, oxidative stress, and altered cellular bioenergetics may amplify neuroimmune effects on neuronal development and synaptic plasticity [73,74,75,119,168,169,170,171,172,173]. Similarly, the gut microbiome may modulate immune maturation, cytokine production, blood-brain barrier dynamics, and neurodevelopmental signaling through the gut-immune-brain axis [100,101,142]. These interacting pathways are summarized in Figure 7. (Figure 7 illustrates a specific microglial and synaptic component within the broader neuroimmune cascade described in Figure 5.)

Recent work continues to support associations between neuroimmune signaling and circuit maturation in autism, with emerging studies highlighting how cytokine-dependent microglial activation may influence synaptic refinement and network connectivity during critical developmental windows [174,175,176,177,178,179,180].

### 12.1. Sex Differences in Neuroimmune Development

Sex differences represent an important dimension of neuroimmune development in ASD. Autism spectrum disorder shows a marked male bias, with males diagnosed more frequently than females [54,107]. This disparity suggests that sex-specific biological mechanisms may influence vulnerability to neurodevelopmental perturbation.

Sexual dimorphism in immune function is well established, with differences influenced by sex chromosomes, sex steroids, and immune signaling pathways [5,104]. Microglia also exhibit sex-dependent patterns of density, morphology, activation, and developmental timing, which may influence synaptic pruning and circuit formation [102,181]. Consequently, similar immune perturbations may produce different effects on neural maturation depending on sex, developmental stage, and hormonal context.

Genetic mechanisms may further contribute to sex differences in ASD risk. Several genes involved in immune regulation and synaptic development are located on the X chromosome, and the female protective effect has been proposed as one explanation for differential vulnerability [9]. Immune signatures may also differ across males and females with ASD, with implications for biomarker development and individualized intervention strategies [6,43].

### 12.2. Limitations and Generalizability

Several limitations should guide the interpretation of the framework advanced here. First, ASD is profoundly heterogeneous, and neuroimmune dysregulation is unlikely to represent a universal mechanism across all presentations. The model is therefore best understood as subgroup-relevant and developmentally contingent.

Second, much of the mechanistic evidence derives from animal models of maternal immune activation, postmortem studies, or peripheral immune markers. These approaches provide important biological insight but cannot be directly interpreted as evidence of causal mechanisms in living human neurodevelopment. Immune markers may also reflect state-dependent processes rather than stable trait characteristics.

Third, developmental timing constrains interpretation. Immune influences during prenatal, perinatal, early postnatal, and later developmental periods may differ substantially. Findings from one developmental stage may not generalize to another. Similarly, sex differences require more systematic integration into neuroimmune ASD research.

Finally, because this is a narrative and integrative review, conclusions depend on the available literature and should be interpreted as a framework for future investigation rather than a definitive causal account. Accordingly, the mechanisms described here are best understood as probabilistic and developmentally contingent, reflecting interactions among multiple biological systems rather than a single deterministic pathway. The model complements, rather than replaces, genetic, environmental, metabolic, and systems-level accounts of ASD.

### 12.3. Future Directions

Future work should clarify the molecular pathways through which cytokines such as IL-6, IL-17A, IL-37, and IL-38 may influence synaptic pruning, myelination, neuronal connectivity, and circuit-level function [11,47,182]. Studies combining molecular neuroscience, immunology, developmental neurobiology, transcriptomics, and neuroimaging will be essential for linking immune signaling to neural outcomes.

Microglial function remains a particularly important target for future research, given its role in synapse elimination, inflammatory signaling, and circuit refinement [98,103]. Future studies should determine how microglial states vary across developmental time, sex, immune phenotype, and ASD subtype, and how these variations relate to connectivity findings in ASD [48,167].

Additional priorities include clarifying immune-metabolic interactions, gut-immune-brain mechanisms, genomic and transcriptomic contributions, and biomarkers for stratification [6,7,73,74,75,95,100,101,142,170,177,183,184,185,186,187,188,189]. Longitudinal studies that combine immune profiling, microbiome assessment, neuroimaging, behavioral characterization, and developmental timing will be especially important.

### 12.4. Implications for Developmental Intervention

The neuroimmune framework has implications for intervention, but these implications must be interpreted cautiously. Immune-related mechanisms may help define biologically meaningful ASD subgroups with differential responsiveness to intervention. Potential targets include cytokine signaling, gut-immune interactions, blood-brain barrier dynamics, glial activity, and neuroinflammatory processes. However, such approaches remain largely investigational and should not be generalized across the autism spectrum.

Rather than suggesting a single therapeutic pathway, the framework supports mechanism-informed and developmentally sensitive strategies. Interventions may need to be stratified according to developmental stage, immune phenotype, biological context, and neural systems involvement. Developmentally appropriate sensory, motor, autonomic, behavioral, and environmental interventions may influence neuroimmune outcomes indirectly by shaping neuronal activity, stress regulation, metabolic demand, and circuit stabilization.

A major translational goal is therefore the development of biomarker-informed models that link immune signatures to developmental trajectories and intervention responsiveness. Stratification approaches may integrate cytokine profiles, microbiome composition, neuroinflammatory markers, metabolic measures, and behavioral phenotyping. Such models may help identify which individuals are most likely to benefit from interventions targeting inflammatory signaling, gut-brain axis integrity, or broader neurodevelopmental regulation.

## 13. Conclusions

ASD is best understood as a heterogeneous neurodevelopmental condition arising from interactions among genetic susceptibility, immune signaling, environmental exposure, metabolic regulation, and neural circuit development. The evidence reviewed here is consistent with a developmental neuroimmune cascade model in which immune processes may influence neurodevelopment through effects on cytokine signaling, microglial activation, synaptic pruning, barrier function, myelination, neurotransmitter balance, and large-scale network maturation.

This model does not propose immune dysfunction as a singular cause of ASD. Rather, it frames neuroimmune processes as developmentally timed modulators of neural maturation that may be especially relevant in biologically defined subgroups. Such a perspective may help explain why immune findings in ASD are variable, why biomarkers remain inconsistent, and why intervention responses may differ across individuals. Within this framework, immune signaling is best understood not as an isolated driver but as a modulatory component within a multilevel developmental system in which timing, genetic architecture, and environmental input jointly shape developmental trajectories.

Framing timing as an important organizing principle may help shift the study of development from descriptive chronologies to mechanistic accounts of how functional structure emerges. This perspective not only integrates motor, cognitive, and social domains within a unified framework but also may open new directions for experimentally probing the temporal foundations of human development.

Future progress will require longitudinal, stratified, and mechanistically grounded research linking immune profiles to developmental timing, neural circuitry, behavioral outcomes, and treatment responsiveness. Integrating neuroimmunology with developmental neuroscience provides a framework for examining relationships between biological heterogeneity and developmental dynamics, offering a basis for testable models of ASD pathophysiology and early identification.

## Figures and Tables

**Table 1:** Major gene categories implicated in autism spectrum disorder and their functional relevance.

Gene Category	Representative Genes	Primary Functional Role	Relevance to ASD Pathophysiology
Synaptic structure and function	SHANK3, NRXN1, NLGN3, NLGN4	Synapse formation, stabilization, and signaling	Disruption leads to impaired synaptic connectivity and altered network organization.
Excitation-inhibition balance	GABRB3, GAD1, GRIN2B, SCN2A	Regulation of inhibitory and excitatory neurotransmission	Imbalance contributes to altered cortical excitability and information processing.
Chromatin remodeling and transcription	CHD8, MECP2, ADNP	Gene expression regulation during development	Affects broad neurodevelopmental programs and neuronal differentiation
Cell adhesion and neuronal migration	CNTNAP2, L1CAM, RELN	Neuronal positioning, migration, and connectivity	Leads to atypical cortical organization and connectivity patterns
Immune-related and inflammatory pathways	HLA region genes, C4A, C4B	Immune signaling and complement activation	Implicated in neuroinflammation and synaptic pruning abnormalities
Intracellular signaling pathways	PTEN, TSC1, TSC2	Cell growth, proliferation, and synaptic plasticity	Dysregulation affects neuronal size, connectivity, and network stability

**Table 2:** Representative evidence linking immune mechanisms to neurodevelopmental and network-level alterations in autism spectrum disorder.

Study	Immune Component	Immune Component	Key Findings	Level of Mechanism
Estes & McAllister [3]	Review	Immune dysregulation in ASD	Foundational evidence linking immune activation to ASD risk	Integrative
Choi et al. [11]	Animal (maternal immune activation)	IL-17A signaling	Maternal IL-17A alters cortical development and induces ASD-like behaviors	Peripheral/ Developmental
Li et al. [36]	Human (ASD cohort)	Neuroimmune-associated structural changes	Insular surface area alterations linked to social impairment	Network-level
Zielinski et al. [37]	Human (longitudinal imaging)	Network development	Altered maturation of cingulate-insular networks in ASD	Network-level
Otero & Antonson [51]	Review/mechanistic synthesis	Maternal immune activation, IL-17A, microglia	Integrates viral, microbial, and immune pathways influencing neurodevelopment	Integrative
Careaga et al., 2010 [52]	Human / review	Neuroinflammation	Early evidence of immune involvement and inflammatory markers in ASD	Central (neuroinflammatory)
Theoharides et al. [53]	Review	Mast cells, BBB permeability	Mast cell mediators may disrupt BBB and influence neuroinflammation	Peripheral → Barrier
Al Rasbi et al. [54]	Review	IL-37, IL-38	Anti-inflammatory cytokines may regulate neuroinflammatory balance in ASD	Peripheral regulatory
Di Gesù & Buffington [55]	Review	Gut–immune–brain axis	Microbiome influences neurodevelopment via immune signaling pathways	Integrative
Huang et al. [56]	Review	Mast cells in neurovascular function	Mast cells contribute to neurovascular and inflammatory processes	Peripheral → Barrier

→ indicates directional influence or interaction between peripheral processes and barrier function.

## Data Availability

No new data were created or analyzed in this study. Data sharing is not applicable to this article. During the preparation of this study, the authors used ChatGPT-5 for the purposes of assisting in the preparation of the graphic material. The authors have reviewed and edited the output and take full responsibility for the content of this publication.
